# RPocket: an intuitive database of RNA pocket topology information with RNA-ligand data resources

**DOI:** 10.1186/s12859-021-04349-4

**Published:** 2021-09-08

**Authors:** Ting Zhou, Huiwen Wang, Chen Zeng, Yunjie Zhao

**Affiliations:** 1 Department of Physics, Institute of Biophysics, Central China Normal University, Wuhan, 430079 China; 2 Department of Physics, George Washington University, Washington, DC 20052 USA

**Keywords:** Pocket database, RNA-ligand interaction, Structure prediction, Drug discovery

## Abstract

**Background:**

RNA regulates a variety of biological functions by interacting with other molecules. The ligand often binds in the RNA pocket to trigger structural changes or functions. Thus, it is essential to explore and visualize the RNA pocket to elucidate the structural and recognition mechanism for the RNA-ligand complex formation.

**Results:**

In this work, we developed one user-friendly bioinformatics tool, RPocket. This database provides geometrical size, centroid, shape, secondary structure element for RNA pocket, RNA-ligand interaction information, and functional sites. We extracted 240 RNA pockets from 94 non-redundant RNA-ligand complex structures. We developed RPDescriptor to calculate the pocket geometrical property quantitatively. The geometrical information was then subjected to RNA-ligand binding analysis by incorporating the sequence, secondary structure, and geometrical combinations. This new approach takes advantage of both the atom-level precision of the structure and the nucleotide-level tertiary interactions. The results show that the higher-level topological pattern indeed improves the tertiary structure prediction. We also proposed a potential mechanism for RNA-ligand complex formation. The electrostatic interactions are responsible for long-range recognition, while the Van der Waals and hydrophobic contacts for short-range binding and optimization. These interaction pairs can be considered as distance constraints to guide complex structural modeling and drug design.

**Conclusion:**

RPocket database would facilitate RNA-ligand engineering to regulate the complex formation for biological or medical applications. RPocket is available at http://zhaoserver.com.cn/RPocket/RPocket.html.

**Supplementary Information:**

The online version contains supplementary material available at 10.1186/s12859-021-04349-4.

## Background

RNA regulates a variety of biological functions by interacting with other molecules. It is currently recognized that more than 70% of the human genome is transcribed into non-coding RNAs [1]. In contrast, 1.5% of the human genome encodes proteins, and only 0.05% of the human genome has been identified as protein-targeted for drug development. A human probably produces more than 15,000 long non-coding RNAs [1]. Thus, even a tiny part of these non-coding RNAs may eventually prove to be disease-related drug targets. For example, the combination of HIV tat RNA with acetyl promazine can inhibit Tat-TAR interaction [2]. Besides, riboflavin exhibits antibacterial properties by targeting flavin RNA riboswitch [3]. Similarly, a very recent study shows the nucleotide analog inhibitors in one essential molecule for the pathogenesis of COVID-19 by binding with virus-dependent RNA polymerase [4]. Thus, it is believed that RNA is more widely involved in the various regulatory processes.

At present, some experimental methods can determine the RNA-ligand structure. Unfortunately, the flexible RNA molecules are challenging to be well-crystallized and determined by X-ray crystallography. Besides, electron microscopy is expensive and time-consuming. The available RNA-ligand experimental structures are few (572 structures on February 19, 2020) due to these technical limitations. Some computational methods can predict the RNA and RNA-ligand structures by homologous fragment modeling [5–12], molecular dynamics simulation [13–16], or docking [17–19]. However, it is still challenging to predict the high accurate RNA-ligand structures due to the limited understanding of the structural principles for RNA-ligand binding.

There are several existing RNA-related databases and tools to provide sequence, structure, or interaction information (Additional file 1: Table 1). For example, (1) the structure databases (the PDB, NAD, PDB-Ligand, and R-bind) provide tertiary structure information of RNA-ligand complexes, structure and physicochemical properties of ligand [20–23]; (2) the RNA-ligand experimental databases (the NALDB, SMMRNA, and KDBI) provide the chemical reaction information and kinetic data of the formation of RNA-ligand [24–26]; (3) RNA docking datasets and tools (the RRDB, HNADOCK, DrugScoreRNA, and LigandRNA) provide the docking algorithms, scoring functions, and docking benchmarks [17, 27–29]; (4) RNA pocket detection tools (3 V, Caver, and PocketFinder) identify RNA pockets and size of pocket [30–32]. However, the available information in these databases cannot be directly used in the RNA ligand study. The well-analyzed RNA pocket and binding sites are still minimal. Thus, a comprehensive and updated RNA pocket database is urgently needed, especially targeting the pockets in RNA for drug development.

Here, we performed a systematic analysis of 240 pockets from 94 non-redundant RNA-ligand complex structures. We first analyzed the characteristic patterns of secondary structure for all the identified RNA pockets. Then, we introduced RPDescriptor to calculate the pocket topology property quantitatively. Moreover, we performed a statistical analysis of the RNA-ligand interaction features. Our results suggest that some charged interaction pairs might provide the long-range steering force to bring the RNA and ligand together. Then, the short-range interactions optimize and stabilize the binding. The different scales of structural topology characteristics may improve the RNA structure prediction and RNA-related drug design. We also developed one user-friendly bioinformatics tool, RPocket, to facilitate ligand design or RNA engineering to regulate the complex formation for biological or medical applications.

## Construction and content

**Fig. 1:**
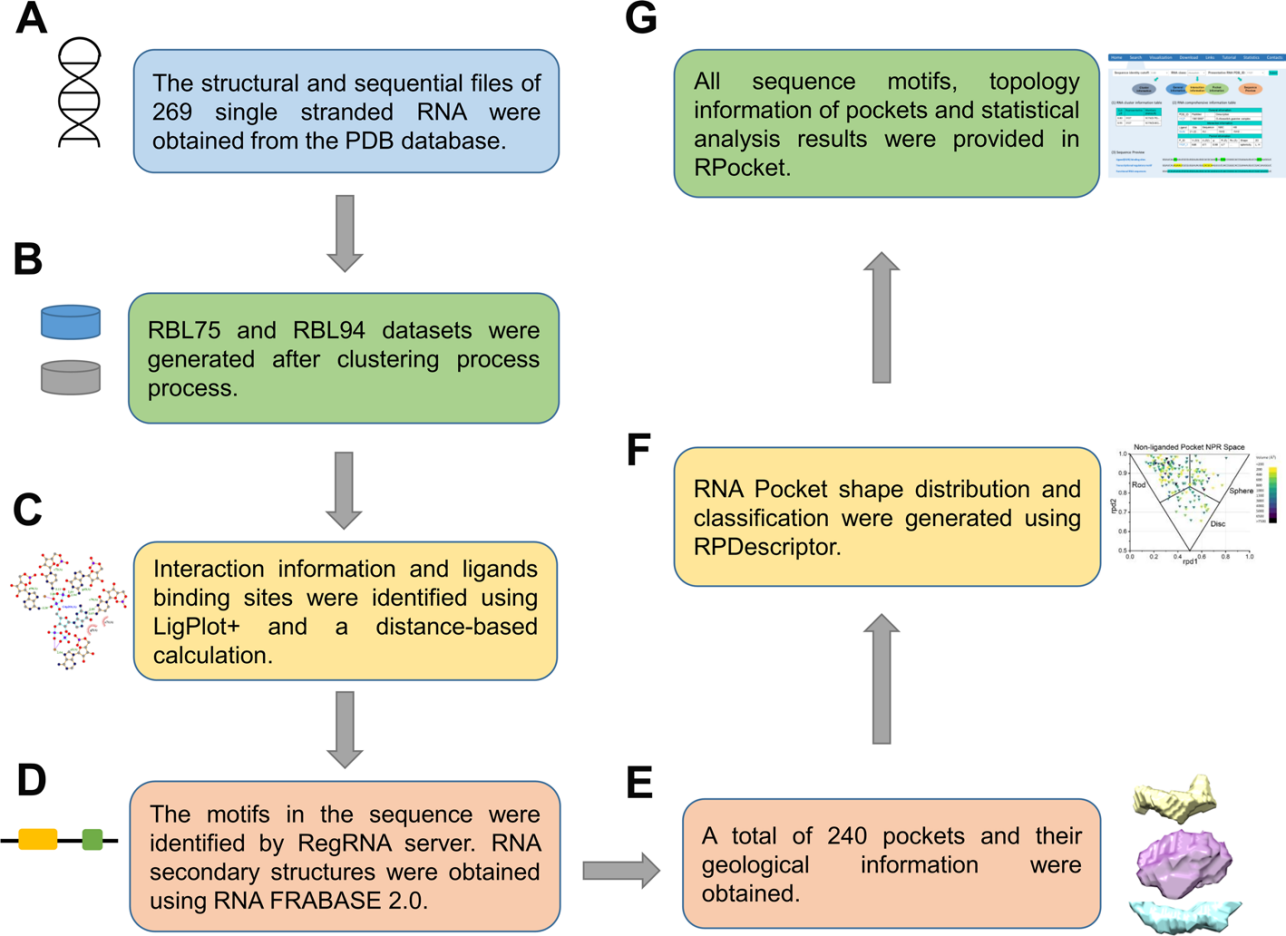
The workflow of the RPocket database construction. **A** 269 RNA-ligand structures were used for analysis. **B** To acquire the non-redundant dataset, we performed the sequence alignment for the 269 structures using the CD-Hit server. We have used two identity cutoffs (0.80 and 0.95) to get relatively loose and more strict non-redundant datasets: RBL75 and RBL94 (75 and 94 clusters for 0.80 and 0.95 sequence identity cutoffs). **C** Interaction information and ligands binding sites were identified using Ligplot + and a distance-based calculation. **D** The functional motifs were identified by the RegRNA program. The RegRNA identifies the RNA motifs by integrating regulatory RNA motifs from the published literature and RNA motif databases. **E** The pockets were detected by the 3 V server using the rolling probe method. **F** RNA pocket shape distribution and classification were generated using RPDescriptor. **G** The ligands functional groups, hydrogen bond and non-bond interactions, the secondary structure patterns, and pocket topology information were calculated and provided in the RPocket server

For biologists to better access the information of RNA pocket, we established a user-friendly online database: RPocket. RPocket contains 240 pocket information of 94 RNA-ligand complex structures (non-redundant). A workflow of constructing the RPocket database is shown in Fig. 1.

(A) The PDB structural files and sequence FASTA files of 1448 RNAs were extracted using the REST API advanced search interface in the Protein Data Bank before February 19, 2020 [33]. Here, we only considered the single-strand RNA molecules with ligands (remaining 298 entries). Then, we removed the short (less than ten nucleotides) and highly complex (more than 500 nucleotides) RNAs. If the RNA has several NMR structures, the first structural model is selected. There are remaining 269 RNA-ligand structures after this screening step.

(B) To acquire the non-redundant dataset, we performed the sequence alignment for the 269 structures using the CD-Hit server [34]. We have used two identity cutoffs (0.80 and 0.95) to get relatively loose and more strict non-redundant datasets: RBL75 and RBL94 (75 and 94 clusters for 0.80 and 0.95 sequence identity cutoffs) [8, 34, 35]. We performed the RMSD calculations to reflect the divergence between the representative and other structures in each cluster [36]. All the representative structures in two non-redundant datasets and the RMSD between representative and class members can be downloaded on the website. For example, one cluster in the non-redundant dataset has 24 RNA-ligand complexes. The representative structure is guanine riboswitch (PDB code: 3FO4). We calculated the RMSDs between 3FO4 and all other RNA-ligand complexes. The RMSDs of 0.30 ± 0.19 Å show that the RNAs in the cluster are highly similar (Additional file 1: Fig. 1). Here, we analyzed the 94 representatives in the RBL94 to obtain the RNA-ligand structural principles.

(C) We identified the RNA-ligand binding sites using a distance-based calculation. A nucleotide is considered one binding site if the distance is less than 4 Å between the RNA and ligand. The detail interactions were generated using Ligplot + with the HBPLUS program [37, 38]. The Ligplot + can provide the hydrogen bond and non-bond contacts between RNA and ligands at the atomic level.

(D) The functional motifs were identified by the RegRNA program [39]. The RegRNA identifies the RNA motifs by integrating regulatory RNA motifs from the published literature and RNA motif databases. The functional motifs can be divided into 12 categories: motifs in transcriptional, Pre-mRNA, translational, UTR motifs, mRNA degradation elements, RNA cis-regulatory elements, RNA editing sites, riboswitches, RNA structural patterns, functional RNA sequences, RNA-RNA interaction regions, and user-defined motifs. In addition, the secondary structure units of stacking bases, interior loop, bulge loop, hairpin loop, multibranch loop, and pseudoknot were identified and generated using RNA FRABASE 2.0 [40–42]. All the identified functional motifs can be downloaded on the RPocket website.

(E) The pockets were detected by the 3 V server using the rolling probe method [30, 43–45]. The volume and surface area were calculated by rolling two virtual probes (a shell probe and a solvent probe) around the van der Waals surface [30, 43–46]. We used the default radius value (10 Å for shell probe radius and 3 Å for solvent probe radius) to extract the RNA pockets.

(F) We developed RPDescriptor (**R**NA **P**ocket **D**escriptor) to calculate the pocket geometric characteristics for RNA molecules. RPDescriptor can generate two descriptors based on Normalized Principal Moments of Inertia Ratios (NPRs) [47]. The shape of the RNA pocket can be visually displayed on an isosceles triangle by projecting the two descriptors ($rpd_{1}$ and $rpd_{2}$) onto the two-dimensional plane. We defined a shape similarity score $s_{i}$ that allows pockets to be classified quantitatively.

(G) The ligands functional groups, hydrogen bond and non-bond interactions, the secondary structure patterns, and pocket topology information were calculated and provided in the RPocket server.

## Utility and discussion

One user-friendly bioinformatics tool for RNA pocket information has been missing. This limitation motivated us to develop the RPocket, a user-friendly web server, to analyze the RNA pockets using a simple graphical user interface. Some advanced features implemented in RPocket are (1) contains 240 pocket information extracted from 94 non-redundant RNA-ligand structures; (2) displays the sequence, secondary structure, and RNA-ligand interaction characteristic patterns; (3) constructs a database with the pocket geometric topology information such as volume, surface area, and shape similarity scores; (4) provides a visualization tool for users to scale and rotate the structure; (5) provides one executable script for users to perform pocket topology analysis. (6) offers the related tools to predict or simulate RNA structures. RPocket web server is a reliable and user-friendly tool and facilitates the RNA pocket study without installing programs locally.

**Fig. 2:**
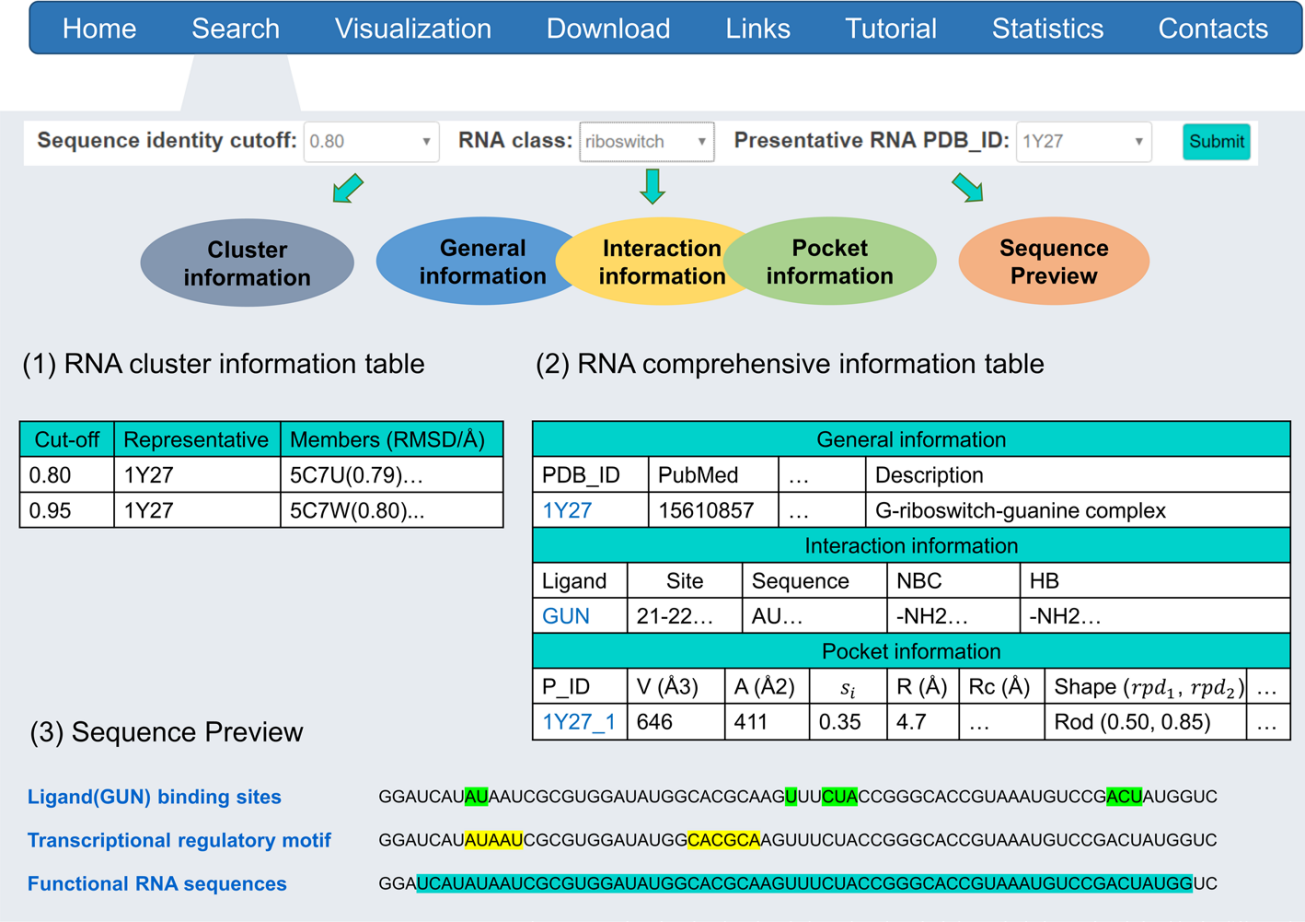
The search module of the RPocket server. The user interface displays the RNA cluster, RNA-ligand interaction, pocket topology, and sequence motif characteristic patterns

RPocket consists of eight modules: Home, Search, Visualization, Download, Links, Tutorial, Statistics, and Contacts. The Home module provides a brief introduction to the RPocket database and navigation to other modules. Users can identify and extract the pocket information using the Search module (Fig. 2). The Search module consists of four parts: a pulldown search box, a summary table of RNA clusters, a table of RNA descriptions, and a sequence preview module. The pulldown search box can identify the RNAs by defining the sequence identity cutoff, RNA class, and PDB ID. The RNA cluster information table shows the RMSD between representative RNA and other members. A comprehensive information table consists of three sections: experiment, RNA-ligand interaction, and pocket geometrical information. Users can click the highlighted links to check the complexes' detailed interaction graph and download the pockets' structure file. The Sequence Preview module shows the ligand-binding sites, sequence motifs with highlighted labels. The combination of topology information of pockets and functional motifs would guide RNA-related drug screening and docking. In the Visualization module, users can upload and investigate the pocket structure. In the Download module, users can download the information of pockets in xlsx format and the structure of pockets in MRC format. The Links module provides the RNA pocket shape classification scripts and other useful links to help RNA-related drug development and vaccine design. The Tutorial module offers the introduction to use the RPocket and the abbreviation for the RPocket database. Some results of data analysis are shown in the Statistics module. The Contacts module provides emails for users to comment or ask questions. More detail about RPocket database utility is described in Additional files (Additional file 1: Section User interface and utility and Figs. 8–11).

## Implementation

### Pocket identification and topology calculation

All the pockets were identified using the rolling probe method by the 3 V program [30, 43–45]. The coordinates of the molecule are superimposed on the cubic grids. The pocket is detected by calculating the translational degrees of freedom of the probe ball. The center of the probe is recorded if the probe contacts with more than two atoms on the molecule [43]. These discrete positions form the rolling boundary of the pocket [44]. The volume and surface area values were calculated by using the discrete volume method. Here we used the tested parameters for RNA pocket detection, which are 10 Å for shell probe radius and 3 Å for solvent probe radius [30]. The effective radius was calculated using the following formula

1 $${\text{r}}_{{{\text{eff}}}} = \frac{{3V_{p} }}{{A_{p} }}$$

where $V_{p}$ and $A_{p}$ represent the volume and surface area. The sphericity (Ψ) was used to measure the similarity between the pocket and sphere using the following formula

2 $$\Psi = \frac{{A_{s} }}{{A_{p} }} = \frac{{\left( {36\pi V_{p}^{2} } \right)^{1/3} }}{{A_{p} }} = \frac{{\pi^{1/3} \left( {6V_{p} } \right)^{2/3} }}{{A_{p} }}$$

$A_{s}$ represents the surface area of a sphere whose volume is the same as the pocket volume,$V_{p}$. The $r_{c}$ is the center of mass to pinpoint the location of the pocket [31, 48].

### Pocket geometric characteristics analysis and classification

**Fig. 3:**
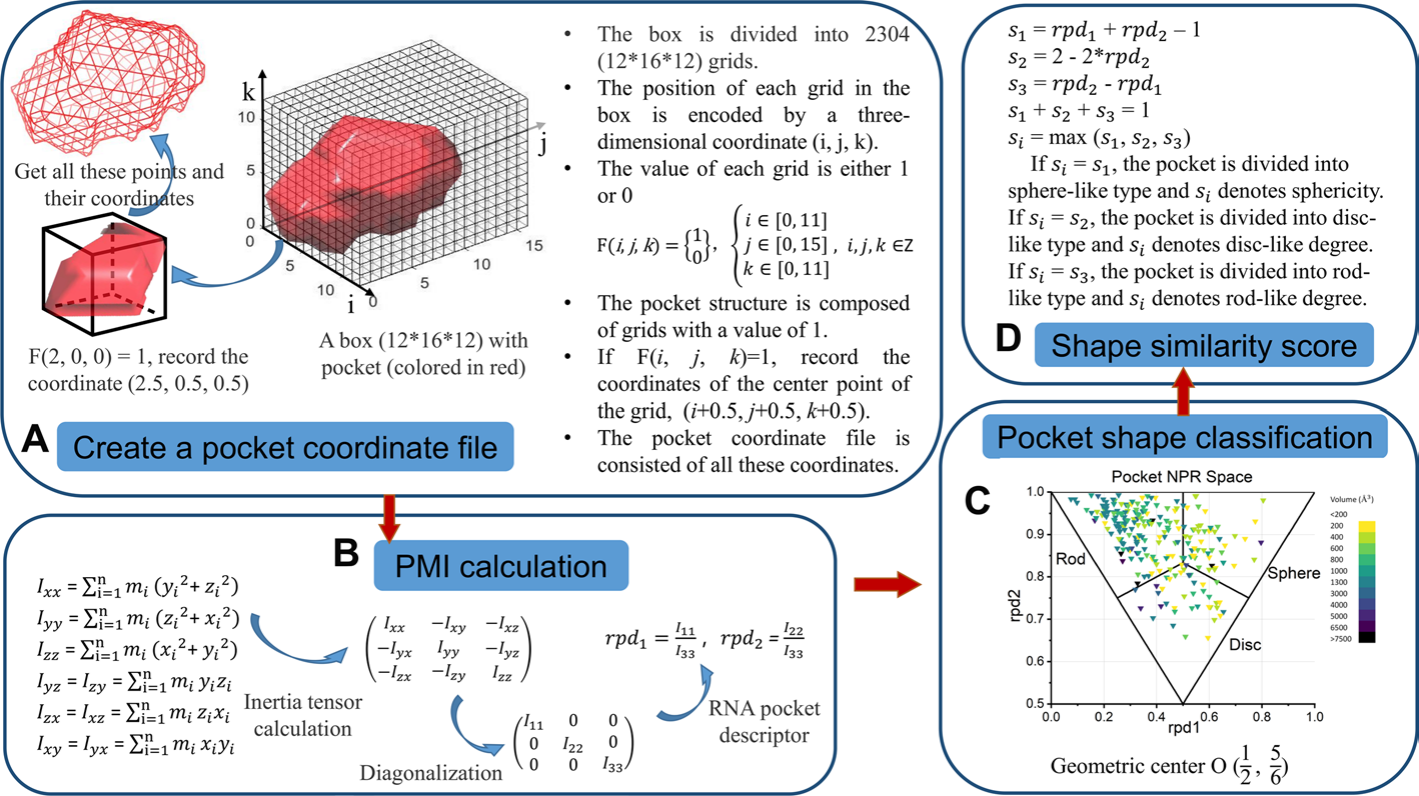
The workflow of the RPDescriptor. The process of the RPDescriptor contains five steps: **A** create the pocket coordinate file from the NetCDF format; **B** principal moment of inertia calculation; **C** generate pocket NPR space graph by projecting the two descriptors onto the two-dimensional plane and classify pocket by connecting the three vertexes of the triangle and the geometric center O; **D** calculate the shape similarity

The geometric characteristics of the RNA pockets were identified by Normalized Principal Moments of Inertia Ratios (NPRs). NPRs display a three-dimensional molecule's shape by projecting two descriptors calculated using the principal moment of inertia (PMI) onto a two-dimensional plane [47]. Previous studies have developed some methods to calculate the PMI for proteins [49]. However, these methods cannot be directly applied for RNA pocket calculation. Thus, we developed RPDescriptor (**R**NA **P**ocket **D**escriptor) to calculate the pocket geometric characteristics for RNA molecules. Figure 3 is the workflow of RPDescriptor taking a particular pocket (1EVV_1) as an example.

The first step is to generate the RNA pocket's coordinate file for NPR analysis (Fig. 3A). The 240 pocket files in MRC format were converted to Network Common Data Format (NetCDF) by Chimera. In NetCDF, a box with length a Å, width b Å, and height c Å is divided into n (n = a*b*c) small grids with a size of 1 Å. A three-dimensional coordinate encodes each grid's position in the box (i, j, k). The values of i, j, k are integers from 0 to a-1, b-1, and c-1, respectively. The value of each grid F(i, j, k) is either 1 or 0. The pocket structure is composed of grids with a value of 1. Since the pocket density map is uniform, we abstract each grid with a value of 1 at the center of the grid with coordinate (i + 0.5, j + 0.5, k + 0.5).

The second step is PMI and RNA pocket topology descriptors calculation (Fig. 3B). The center of mass can be calculated using RPDescriptor. The moment of inertia tensors around the center of mass was further calculated. The PMI ($I_{11}$, $I_{22}$, $I_{33}$) values were obtained in ascending order. Finally, the RNA pocket topology descriptors, $rpd_{1}$ and $rpd_{2}$, are generated using formula (3).

3 $$rpd_{1} = \frac{{I_{11} }}{{I_{33} }},\;rpd_{2} = \frac{{I_{22} }}{{I_{33} }}$$

The third step is to calculate the pocket shape space quantitatively and classify the shape of the pocket (Fig. 3C). The shape can be visually displayed on an isosceles triangle by projecting the two descriptors ($rpd_{1}$ and $rpd_{2}$) onto the two-dimensional plane. The upper left, upper right, lower-middle diagonal points correspond to a standard rod, sphere, or disk shape, respectively. According to calculation, the isosceles triangle's geometric center is O $\left( {\frac{1}{2}, \frac{5}{6}} \right)$. Then, the O point and the three vertices of the triangle are connected. The shape space can be divided into three categories: sphere-, disc-, and rod-like pockets qualitatively.

The fourth step is to calculate the shape similarity score (Fig. 3D). The $s_{1}$ = $rpd_{1}$ + $rpd_{2}$ − 1, $s_{2}$ = 2—2*$rpd_{2}$, and $s_{3}$ = $rpd_{2}$ − $rpd_{1}$ represent the sphere-like, disc-like, rod-like degree of the pocket, respectively [50]. Here, we defined a shape similarity score $s_{i}$ that allows pockets to be classified quantitatively using formula (4). The value of $s_{i}$ is from $\frac{1}{3}$ to 1. For O point, $s_{i}$ = max $\left( {s_{1} = \frac{1}{3},\;s_{2} = \frac{1}{3},\;s_{3} = \frac{1}{3}} \right)$ = $\frac{1}{3}$. For the three vertices, $s_{i}$ = 1. If $s_{i}$ = $s_{1}$, the pocket is divided into the sphere-like type and $s_{i}$ denotes sphericity. If $s_{i}$ = $s_{2}$ or $s_{3}$, the pocket is divided into a disc-like type or rod-like type, and $s_{i}$ denotes disc-like degree or rod-like degree. We observed that the two shape classification methods (qualitative and quantitative) are equivalent.

4 $$s_{1} + s_{2} + s_{3} = 1,\;\;s_{i} = {\text{max(s}}_{{1}} {,}\;{\text{s}}_{{2}} {,}\;{\text{s}}_{{3}} {)}$$

## Results

### Overview of the RNA pockets

We performed a systematic analysis of the 240 RNA pockets extracted from 94 non-redundant RNA-ligand complex structures (Additional file 5: Folder S1). RNAs can fold into various conformations and affect different functions. The representative RNAs include forty-four riboswitches, fifteen aptamer RNAs, seven ribozymes, five tRNAs, four rRNAs, three small RNAs, two xrRNAs, one mRNA, one telomeric RNA, and thirteen other RNAs [51] (Additional file 1: Fig. 2). For example, the RPocket dataset contains 44 riboswitches and 147 riboswitch pockets. The riboswitch RNA can bind small molecules to regulate gene expression through conformational changes. Understanding the riboswitch pocket provides a potential mechanism for the functional changes and solution for antibiotic drug design. To reflect the difference of characteristic analysis on the geometrical shape of pockets, we analyzed all the pockets topology features using NPRs. RNA pockets can be divided into three categories: sphere-like (50), disc-like (39), rod-like (151) pockets (Additional file 1: Fig. 3).

### Topology characteristic of RNA pockets

**Fig. 4:**
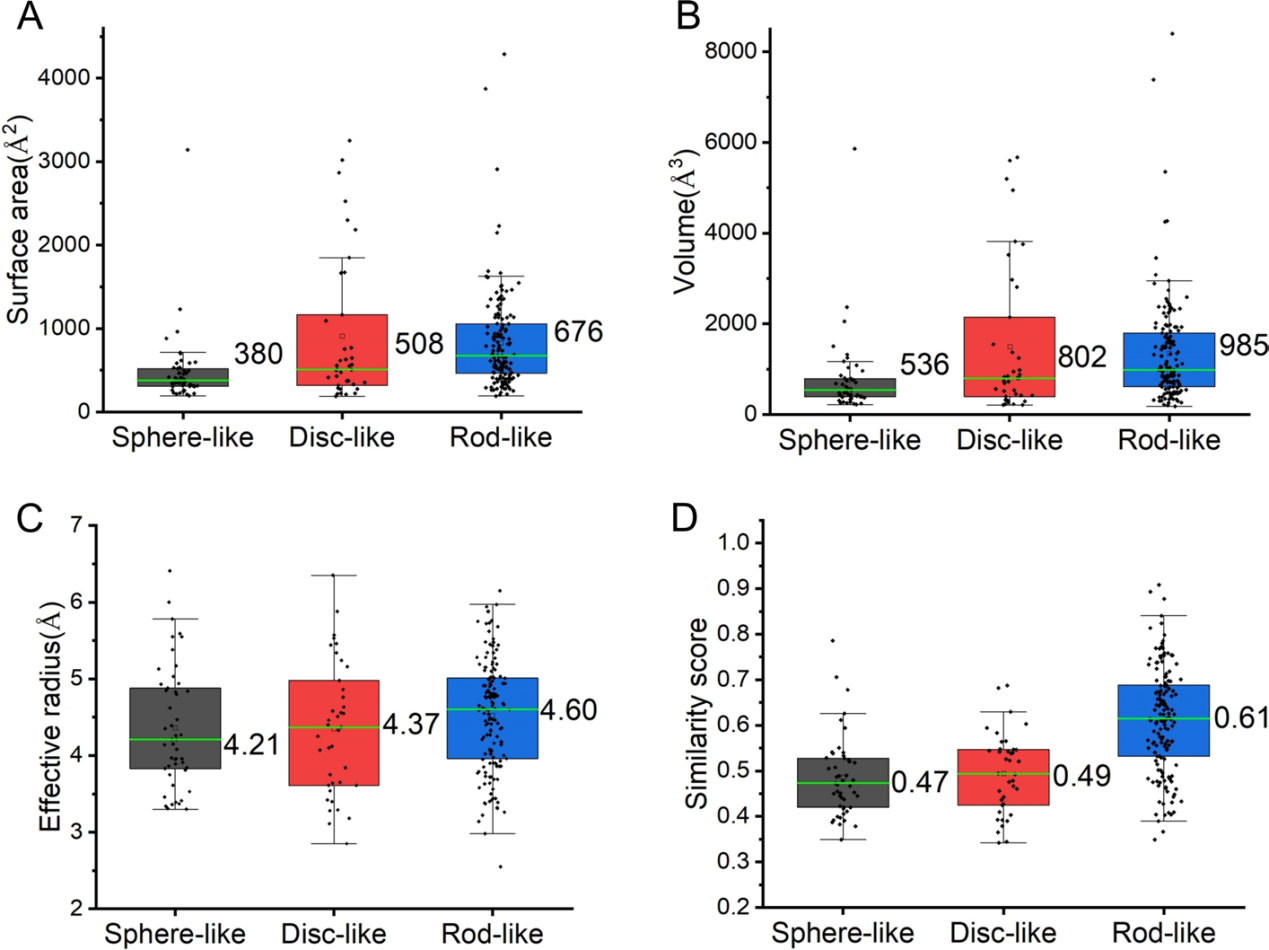
The geometric information distribution of surface area (**A**), volume (**B**), effective radius (**C**), and shape similarity scores (**D**) for each pocket category, respectively. The median values are colored green

The pocket topology characteristic is helpful to identify the small molecules for target-specific binding. We analyzed the topology properties (volume, surface area, and effective radius properties) using a rolling probe method by 3 V program [30]. The mean volume (m) and standard deviation (σ) of all the pockets are 1440.9 ± 2329.4Å^3^. Three large pockets were removed due to their volumes are larger than m + 3σ (Additional file 1: Fig. 4). Then, we calculated the shape similarity scores ($s_{i}$) (Additional file 1: Table S2). Figure 4A–C shows that the rod-like pocket (volume of 985Å^3^, the surface area of 676Å^2^, and effective radius of 4.60 Å) is more extensive than sphere-like (volume of 536Å^3^, the surface area of 380Å^2^, and effective radius of 4.21 Å) and disc-like (volume of 802Å^3^, the surface area of 508 Å^2^, and effective radius of 4.37 Å) pockets. We further analyzed the shape similarity scores to reflect pocket shape quantitatively. The continuous similarity scores are from $\frac{1}{3}$ to 1. Grade 1 indicates a standard shape which is a sphere or disc or rod. Grade $\frac{1}{3}$ suggests a very irregular shape. The shape similarity scores of sphere-like, disc-like, and rod-like pockets are 0.47, 0.49, and 0.61, respectively (Fig. 4D). The results suggest that the RNA pockets with rod-like shapes are typically highly rod-shaped, while the sphere- and disc-like class face the absence of highly spherical and discoid shapes, respectively.

**Fig. 5:**
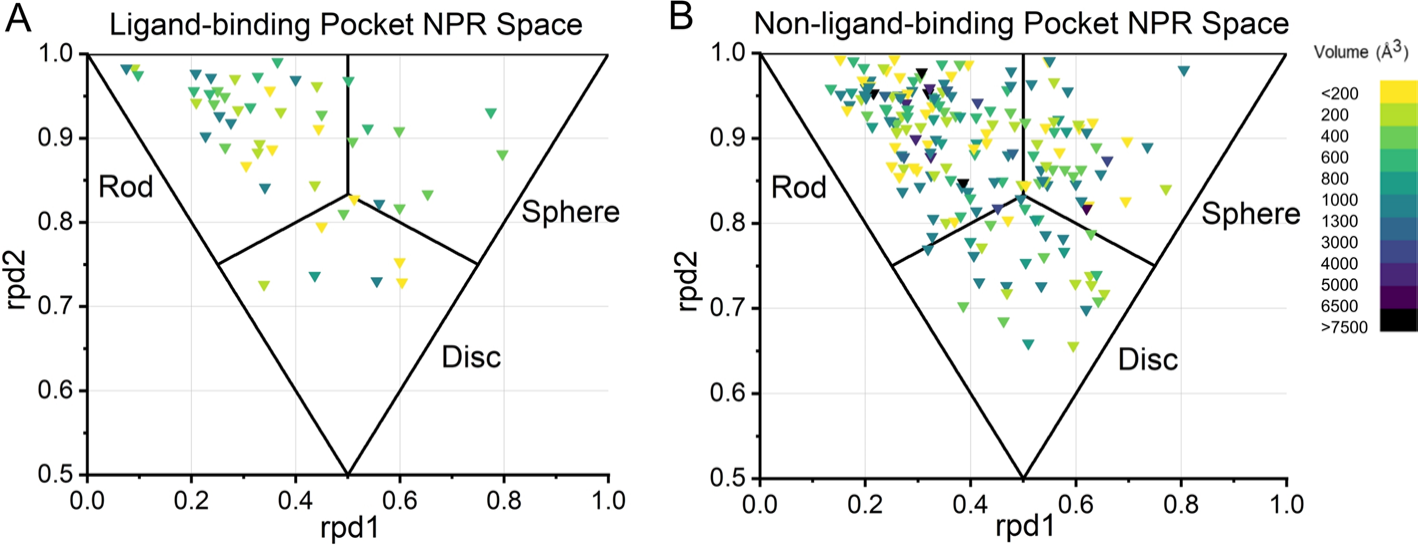
The NPR distribution of ligand-binding and non-ligand-binding pockets. Color code shows the volume size for each pocket

We performed the comparative analysis of the 50 ligand-binding and 190 non-ligand-binding pockets to obtain the topological principle for ligand binding. We classified the RNA pockets based on their geometric shapes using RPDescriptor. There are 9 sphere-like, 8 disc-like, and 33 rod-like pockets in 50 ligand-binding pockets. The geometric shape distribution of 190 non-ligand-binding pockets is similar, which are 41 sphere-like, 32 disc-like, 117 rod-like pockets. To further reflect the geometrical characteristic on shape distributions of ligand-binding and non-ligand-binding pockets, an NPR space distribution graph with pocket-size information was generated. Figure 5 shows that the shape distributions of ligand-binding and non-ligand-binding pockets are similar. We also observed that the location of pockets in RNA are identical. These results emphasize the potential of the non-ligand-binding pocket as a small molecule target. Besides, the loss of globularity with increasing pocket volume both for ligand-binding and non-ligand-binding RNA pockets is consistent with protein pockets, suggesting that RNA can be considered as drug targets like proteins [50]. We further compared the volume and surface area between the ligand-binding and the non-binding pockets (Additional file 1: Fig. 5). It shows most ligand-binding pockets (~ 75%) with a volume between 200 and 2000 Å^3^. The volume and surface area of the ligand-binding pockets (982 Å^3^ median volume and 622 Å^3^ median area) are bigger than non-ligand-binding pockets (803 Å^3^ median volume and 543 Å^3^ median area). The ligand-binding may affect the pocket breathing motions.

### Secondary structure pattern of RNA pockets

**Fig. 6:**
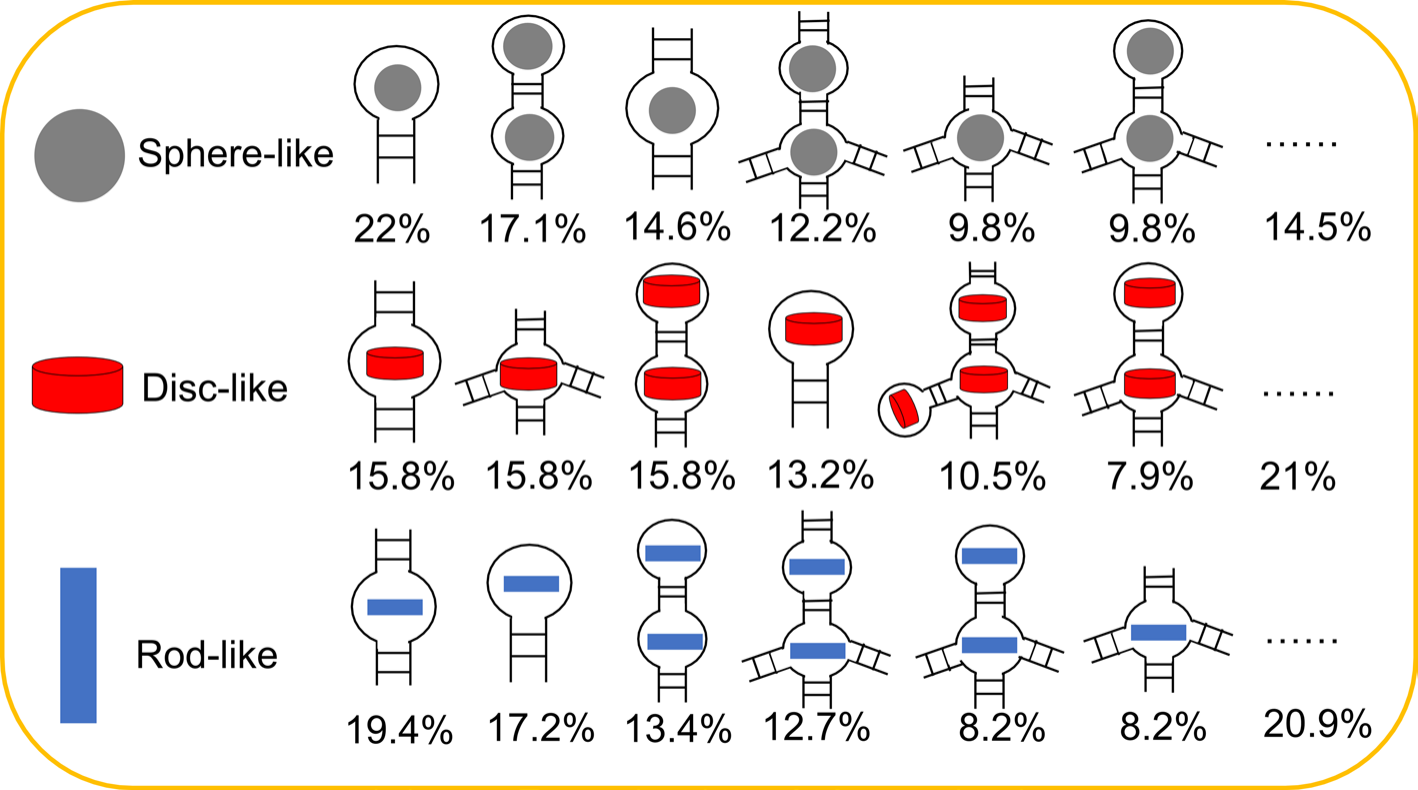
The secondary structure patterns in the RNA pockets. The gray circle represents a spherical-like pocket. The red pie represents a disc-like pocket. The blue rectangle represents a rod-like pocket

The ligand-binding sites usually locate in a specific RNA secondary structure. Binding to the wrong secondary structure may destroy the interactions and the structural stability [52]. Thus, we analyzed the secondary structure distributions for all the RNA pocket binding sites (Additional file 3: Table S2). Here, we focused on the unpaired loop units. There are 10, 11, 15 secondary patterns in the sphere-, disc-, and rod-like pockets. Figure 6 shows that the sphere-like pockets are located in the hairpin loop (22%), internal-hairpin loop (17.1%), internal loop (14.6%), multibranched-internal loop (12.2%), multi-branched loop (9.8%), multibranched-hairpin loop (9.8%), and others (14.5%). The disc-like pockets are observed in the internal loop (15.8%), followed by the multi-branched loop (15.8%), internal-hairpin loop (15.8%), hairpin loop (13.2), internal-multibranched-hairpin-bulge loop (10.5%), multibranched-hairpin loop (7.9%), and others (21%). The rod-like pockets are located in the internal loop (19.4%), hairpin loop (17.2%), internal-hairpin loop (13.4), internal-multibranched loop (12.7%), multibranched-hairpin loop (8.2%), multi-branched loop (8.2%), and others (20.9%). Sphere-like pockets are typically smaller in size than the other two types. This kind of pocket often locates in the hairpin loop with four to five nucleotides [53]. We further counted the numbers of base pairs between the adjacent loops. The results show that the distance of the most adjacent loops are less than six base pairs (86.5%) (Additional file 1: Fig. 6). It is noted that 92.6% of these tandem loops are typically in the same shape pockets.

**Fig. 7:**
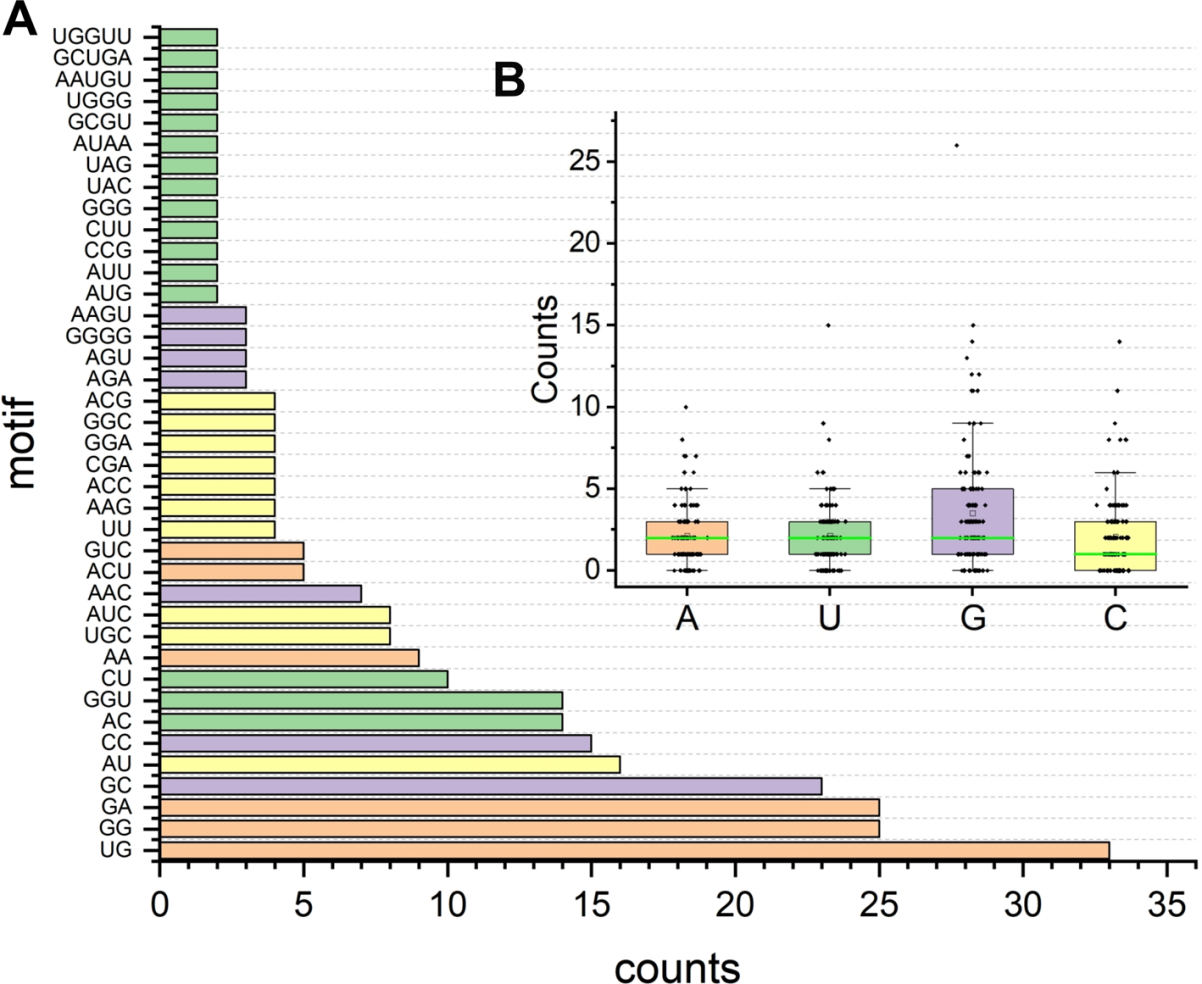
The motif (**A**) and binding site (**B**) distribution of the RNA-ligand structure. The statistical motif analysis is consistent with the confirmed functional sequences. The average distribution of G nucleotides (35.6%) is significantly higher than A (22.1%), C (20.6%), and U (21.7%)

We analyzed the distributions of the nucleotides extracted from the RNA-ligand binding sites (Additional file 4: Table S3). It is noted that the average distribution of G nucleotides (35.6%) is significantly higher than A (22.1%), C (20.6%), and U (21.7%) (Fig. 7B). The nucleotide G is easier to form the hydrogen bond with small molecules. Identifying RNA sequence motif can help us understand the RNA-ligand interactions and function [54]. Thus, we further performed a sequence pattern analysis of the RNA-ligand interaction nucleotides. For example, we consider the continuous symmetric sequence, ‘GU’ and ‘UG’, as the same motif. There are 39 sequence motifs involved in RNA-ligand interactions (Fig. 7A). The sequence motif of ‘GU’ (11.7%), ‘GG’ (8.8%), ‘GA’ (8.8%), ‘GC’ (8.1%), ‘AU’ (5.7%), ‘CC’ (5.3%), ‘AC’ (4.9%), ‘UGG’ (4.9%), ‘CU’ (3.5%), ‘AA’ (3.2%), ‘UGC’ (2.8%), ‘AUC’ (2.8%), ‘AAC’ (2.5%), ‘ACU’ (1.8%), and ‘GUC’ (1.8%) are observed more than five times in all the RNA-ligand interactions. Previous studies have indicated that the motifs ‘GU’, ‘GG’, ‘GA’, ‘GC’ can modulate metal-binding specifically [55]. Some of the sequence patterns have been identified as important motifs for RNA complex formation. For example, the previous study showed that some proteins specifically bind to AR (androgen receptor) mRNA rich in the UC region and play a role in post-transcriptional regulation of AR expression in prostate cancer cells [56]. Besides, the most repeated trinucleotide UGG (14 out of 283) is specifically recognized by Nitrosomonas MazF (a sequence-specific toxin endoribonuclease) and promotes RNA degradation selectively [57].

### Contribution of the short- and long-range interactions

**Fig. 8:**
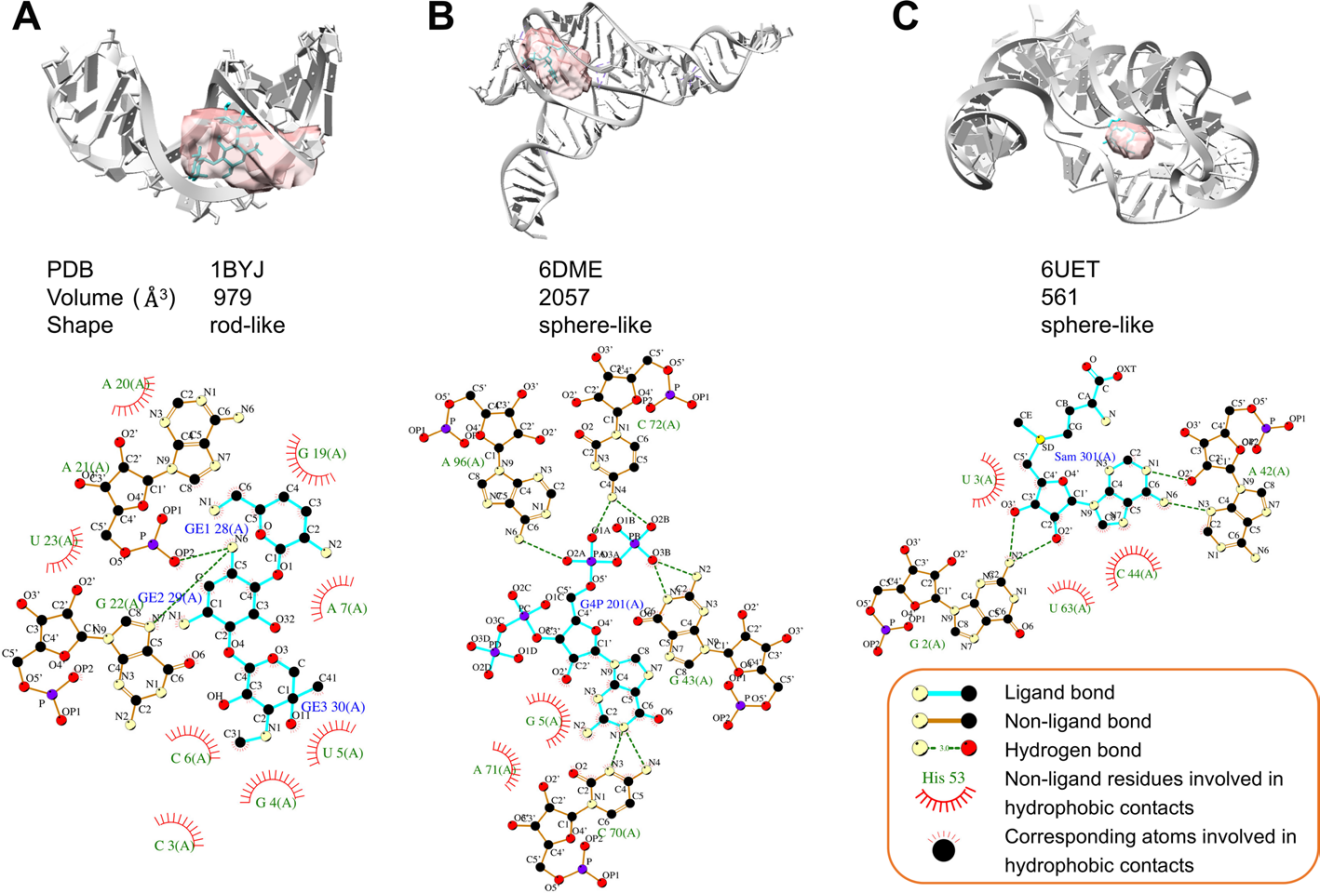
Examples of ligand-binding pockets. **A** The 16S RNA bound to gentamicin C1a (PDB: 1BYJ), **B** the ppGpp riboswitch bound to guanosine tetraphosphate (G4P) (PDB: 6DME), **C** the SAM-IV riboswitch bound to S-Adenosylmethionine (SAM) (PDB: 6UET). The ligands and pockets are colored in cyan and pink. The van der Waals and stacking interactions are emphasized with red arcs. The hydrogen bonds are shown with green dashed lines. The carbon, nitrogen, and oxygen atoms are shown as black, cream, and red spheres

We identified the RNA-ligand interactions and analyzed the interaction patterns using Ligplot + (Additional file 4: Table S3). Figure 8A shows 16S RNA binding with Gentamicin C1a (GE), one of the aminoglycoside antibiotics in a rod-like pocket (volume of 979 Å^3^). There are two hydrogen bonds and eight non-bond interactions involved in the RNA-ligand interactions. It is noted that the hydrogen bonds located the adjacent nucleotides (A21, G22) and eight non-bond interactions dispersed in other parts of the RNA pocket. The other two examples show similar characteristics. The short- and long-range interactions are distributed in different parts of small molecules and stabilize the interaction between RNA and small molecules (Fig. 8B, C). We also analyzed all the ligand functional groups of the 94 representative RNAs involved in hydrogen bond and non-bond interactions (Additional file 1: Fig. 7 and Additional file 6: Folder S2). The results indicate that long-range (polar or electrostatic) interactions bring the ligand and RNA together. Then, the short non-bond interactions optimize the RNA-ligand binding. Besides, we analyzed the size of the pocket and ligand. SAM's volume in space is the smallest, followed by GE, G4P has the biggest size, which is consistent with pocket size. Together, the results suggest two steps for drug screening. First, the size and shape between the RNA pocket and small molecule should be roughly the same. Second, the typically short- and long-interactions should be considered to optimize the RNA-ligand binding.

### Topology pattern improves tertiary structure prediction

**Fig. 9:**
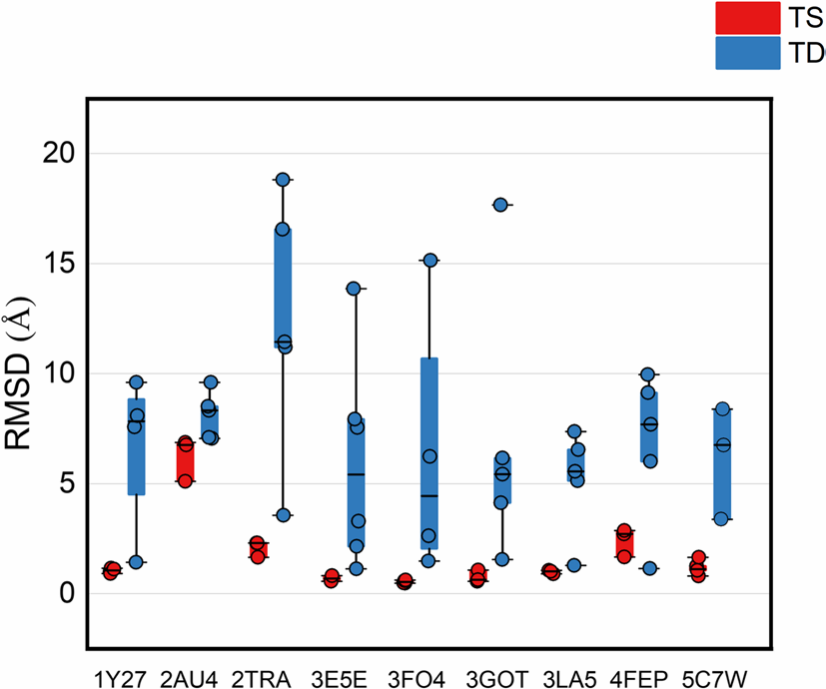
The RMSD values for predicted 3D structures for the tested RNAs. The 3D structures are generated by 3dRNA, RNAcomposer, simRNA, and Vfold3D. We divided the prediction structures into Tandem loops with the Same pocket topology (TS) and Tandem loops with Different pocket topologies (TD). The red dots indicate structures with TS characteristics. These structures (red dots) generally achieve lower RMSD values than the predicted structures with TD characteristics (blue dots)

At present, the structural base pairing and loop elements have been successfully applied to RNA tertiary structure prediction. However, the understanding of the higher-level structural element combinations is still limited. Our results show that 92.6% of the tandem loops (distance less than six base pairs) are typically in the same shape pockets. To test if the higher-level scale of structural elements can identify native-like RNA structures, we ran four popular RNA tertiary structure prediction programs (3dRNA, RNAcomposer, simRNA, and Vfold3D) on the given testing set to build several tertiary structures and evaluated the prediction accuracy (Additional file 1: Fig. 6, Additional file 7: Folder S3). All the tests can be downloaded from our website. We divided the prediction structures into Tandem loops with the Same pocket topology (TS) and Tandem loops with Different pocket topologies (TD). Figure 9 shows the all-atom root-mean-square deviation (RMSD) measured against the native structure. The predicted structure with the TS characteristic shows lower RMSDs (1.71 ± 1.66 Å) while the predicted structure with TD characteristic presents much larger RMSDs (7.23 ± 4.43 Å). The results suggest that the different scales of higher-level topology patterns may improve the RNA tertiary structure prediction.

## Conclusions

In this work, we proposed RPDescritor to calculate the topological properties for RNA pockets quantitatively. The topological information was then subject to RNA-ligand binding analysis by incorporating the sequence and secondary structure information. This new approach takes advantage of both the atom-level precision of the structure and the residue-level tertiary interactions. Together, the results indicate that long-range interactions bring the ligand and RNA together. Then, the short non-bond interactions optimize and stabilize the RNA-ligand binding. We also developed one user-friendly bioinformatics tool, RPocket, to facilitate RNA-ligand engineering to regulate the complex formation for biological or medical applications.

## Supplementary Information

**Additional file 1**. Supplementary material. This file includes the introduction of RPocket, Supplementary material Figure 1–11 and Supplementary material Table 1.

**Additional file 2. Table S1**: RNA-ligand complexes involved in this study.

**Additional file 3. Table S2**: Geometrical information of RNA pockets and secondary structural elements which pocket located.

**Additional file 4. Table S3**: Binding sites of RNA-ligand complexes and functional groups of ligands involved in interaction with RNA.

**Additional file 5. Folder S1**: The structure of all RNA pockets.

**Additional file 6. Folder S2**: Interaction info of RNA-ligand complexes.

**Additional file 7. Folder S3**: Nine other experimental structures and their modeling structures and pockets.

**Additional file 8. Folder S4**: RPDescriptor program code for shape classification of RNA pockets.

## Data Availability

All the supplementary data and materials can be downloaded from the homepage of the RPocket at http://zhaoserver.com.cn/RPocket/RPocket.html.

## Declarations

### Declarations

The author declare that they have provided the data and code public accessible.
