# Supplementary material for: RPocket: an intuitive database of RNA pocket topology information with RNA-ligand data resources
Source: BMC Bioinformatics. 2021 Sep 8;22:428. doi: 10.1186/s12859-021-04349-4 (PMC8424408; doi:10.1186/s12859-021-04349-4)
Supplement: Supplementary file 1 — Additional file 1. Supplementary material. This file includes the introduction of RPocket, Supplementary material Figure 1–11 and Supplementary material Table 1. [file 12859_2021_4349_MOESM1_ESM.docx]

**Supplementary Materials**

**RPocket: an intuitive database of RNA pocket topology information with RNA-ligand data resources**

Ting Zhou^1^, Huiwen Wang^1^, Chen Zeng^2^, and Yunjie Zhao^1*^

^1^Institute of Biophysics and Department of Physics, Central China Normal University, Wuhan 430079, China

^2^Department of Physics, George Washington University, Washington, DC, 20052, USA

*Corresponding author

Yunjie Zhao Email: [yjzhaowh@mail.ccnu.edu.cn](mailto:yjzhaowh@mail.ccnu.edu.cn)

**1 The comparison between RPocket and other related databases**

The comparison between RPocket and other related databases is shown in Table 1 [1-16].

**2 An example cluster**

Fig. 1 shows the RMSD calculation results between a representative structure 3FO4 and other members in the cluster.

**3 RNA structure and pocket classification**

RNA structure and pocket classification are shown in Fig. 2-3.

**4 RNA pocket volume distribution**

RNA pocket volume distribution is shown in Fig. 4.

**5 The size distribution of ligand-binding and the non-binding pockets**

Fig. 5 shows the volume and surface area of the ligand-binding pockets and non-ligand-binding pockets.

**6 Two adjacent loops**

Base pairs number statistics between two adjacent loops and three examples of the secondary structure of tandem loops are shown in Fig. 6.

**7 The statistical result of the interaction group**

A hierarchical map of functional groups of ligands for interactions is shown in Fig. 7.

**8 User interface and utility**

RPocket provides a user-friendly online server. As shown in Fig. 8, in RPocket, we offered eight modules: Home, Search, Visualization, Download, Links, Tutorial, Statistics, and Contacts. The detailed information for each module is as follows. Each section contains sufficient help materials to ensure easy use without any prerequisites.

**8.1** **Home module**

The RPocket Home module (Fig. 8) briefly introduces the RPocket database content and function. It also provides navigation to other modules.

**8.2 Search module**

The Search module (Fig. 9) consists of three parts: a pulldown search box, a summary table of RNA clusters, and RNA descriptions. The cluster table shows the cluster conclusion of two sequence identity cutoff, 0.80 (75 clusters) and 0.95 (94 clusters), respectively, and provides RMSD between presentative RNA and members for each cluster. Users can expand or collapse tables to see the content. The RNA description table is the result of the presentative RNAs classification based on their function, and it provides users with the class name, function description, and members in the class. The two tables are designed to help users retrieve information using the pulldown search box easily. Users can select the object by sequence identity cutoff, RNA class, and presentative RNA PDB ID in the pulldown search box. For example, we fill in ‘0.80’, ‘riboswitch’, ‘1Y27’ in the three drop-down menus, respectively, and it means we select an RNA structure whose PDB ID is 1Y27 and belongs to the riboswitch of the RLB75 dataset. Then a new page displays (Fig. 10), which shows the detailed information for 1Y27. (1) A cluster table was made for showing the RMSD between presentative RNA (1Y27) and other members in this cluster. (2) A comprehensive information table consists of three sections: general information section provides the general information of 1Y27 structure including PDB ID, PubMed, method, resolution, organism, class, Nts, and a brief description; the interaction information section provides the sites and sequences in RNA which binds to ligands, and the functional groups in ligands which interact with RNA; the pocket information section contains the geometrical information (Volume, Area, Sphericity, Effective radius, Centroid, rpd1, rpd2, shape similarity score, shape and the secondary structural elements the pocket located), and the secondary structural elements which pocket located in. Users can click the blue ligand name or pocket ID in the table to check the detailed interaction graph of complexes (Fig. 11A) and download the structure file of pockets in MRC format. (3) The third section is ‘Sequence Preview’, which shows the ligand-binding sites, and motifs or function sites in a sequence by adding shadows in different colors. Users can click the shadow part for motifs or function sites to enter the RegRNA server for details (Fig. 11B).

**8.3 Visualization module**

In the visualization module, users can upload and investigate the pocket structure (Fig. 11C). The pocket structure will be visualized in four representations: ‘spacefill’, ‘wire’, ‘ball&stick’, and ‘cartoon’. The key residues can be highlighted in different colors. Users can scale and rotate the pocket structures. Users can also generate and save the picture.

**8.4 Download module**

In the “Download module”, users can download the RNA pocket files (MRC and NetCDF format), the RNA pocket topology information (xls format), RNA-ligand interaction information (xlsx format and RAR format), nine predicted structures involved used in structure modeling section and RPDescriptor program.

**8.5 Links module**

The Links module provides the other useful links of RNA 3D structure resources, sequence alignment, RNA modeling, molecular dynamics, molecular docking, and molecular visualization analysis. These useful websites would be helpful in RNA-related drug development and vaccine design.

**8.6 Tutorial module**

The Tutorial module provides the introduction to use the RPocket and the abbreviation for the RPocket database.

**8.7 Statistics module**

Some statistical analysis was generated, including the motifs of binding sites, ligands functional groups involved in hydrogen bond interactions and non-bond contacts, the binding motifs of pockets on 2D topology, RNA pocket-size distribution, and pocket shape distribution. These statistical results could help researchers in the screening of small-molecule drugs.

**8.8 Contacts module**

The Contacts module provides emails for users to comment or ask questions.

**Figures and Tables**

**
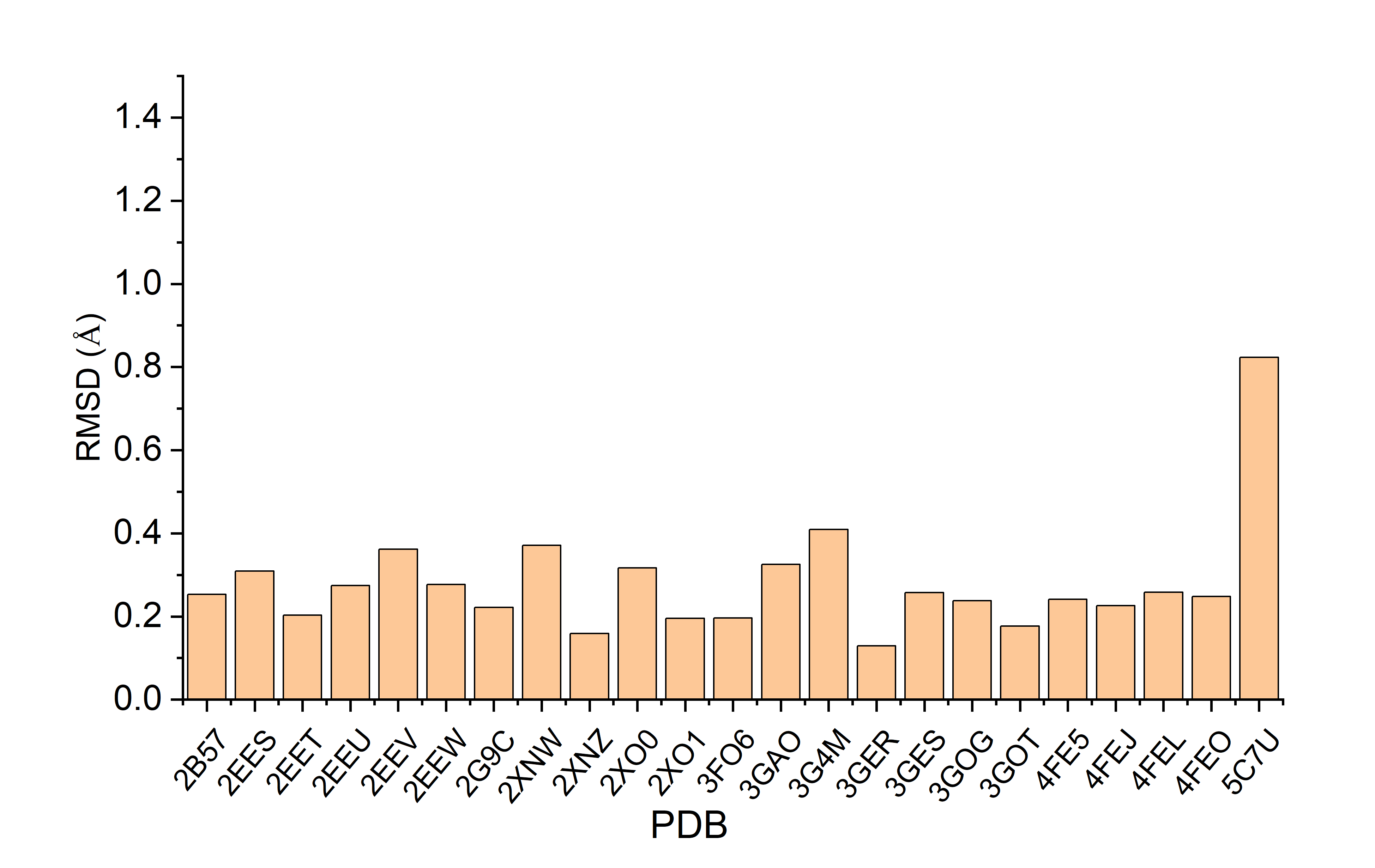
**

**Figure 1.** The RMSDs between the representative and other members of an example cluster.

**
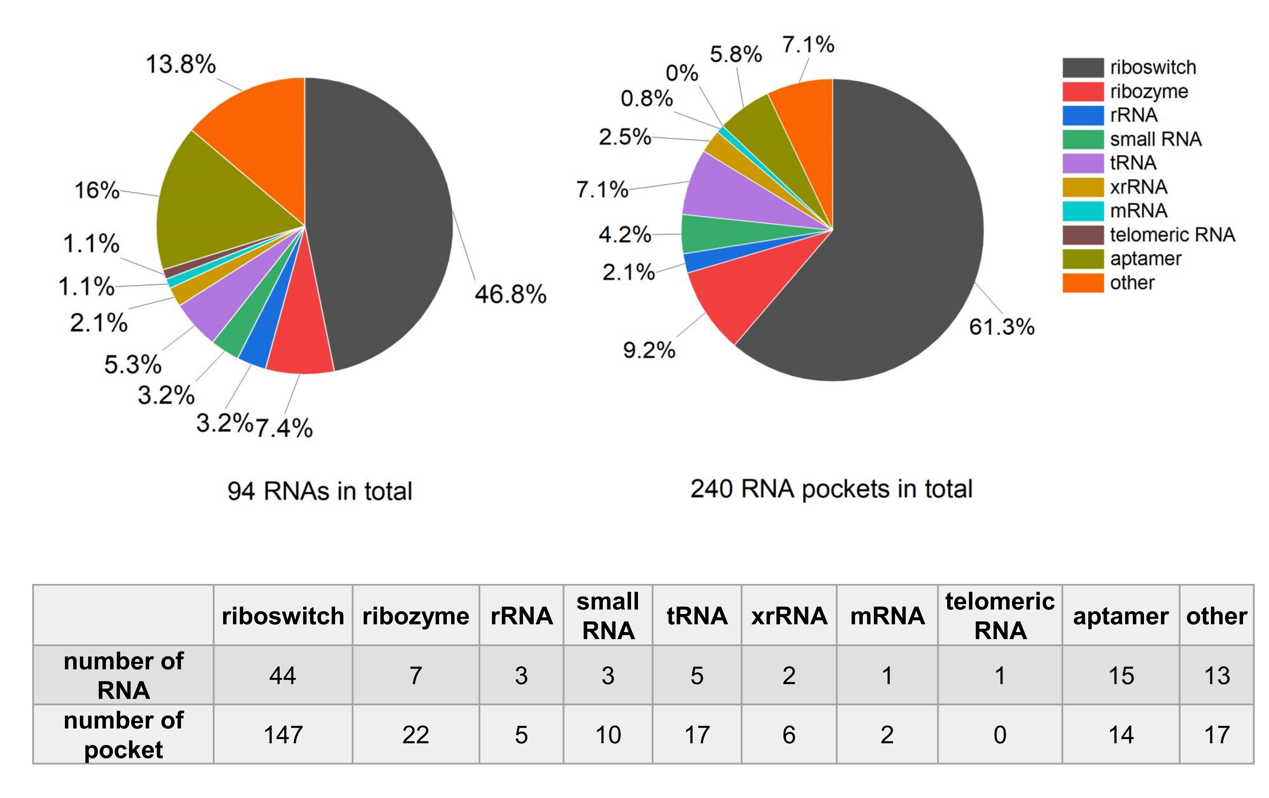
**

**Figure 2.** RNA classifications are based on functions and the pocket distribution for each RNA class. The Pie chart on the left shows the RNA classifications based on functions. The Pie chart on the right shows the pocket distributions for each RNA class. The table shows the numbers of RNAs and pockets for each RNA class.

**
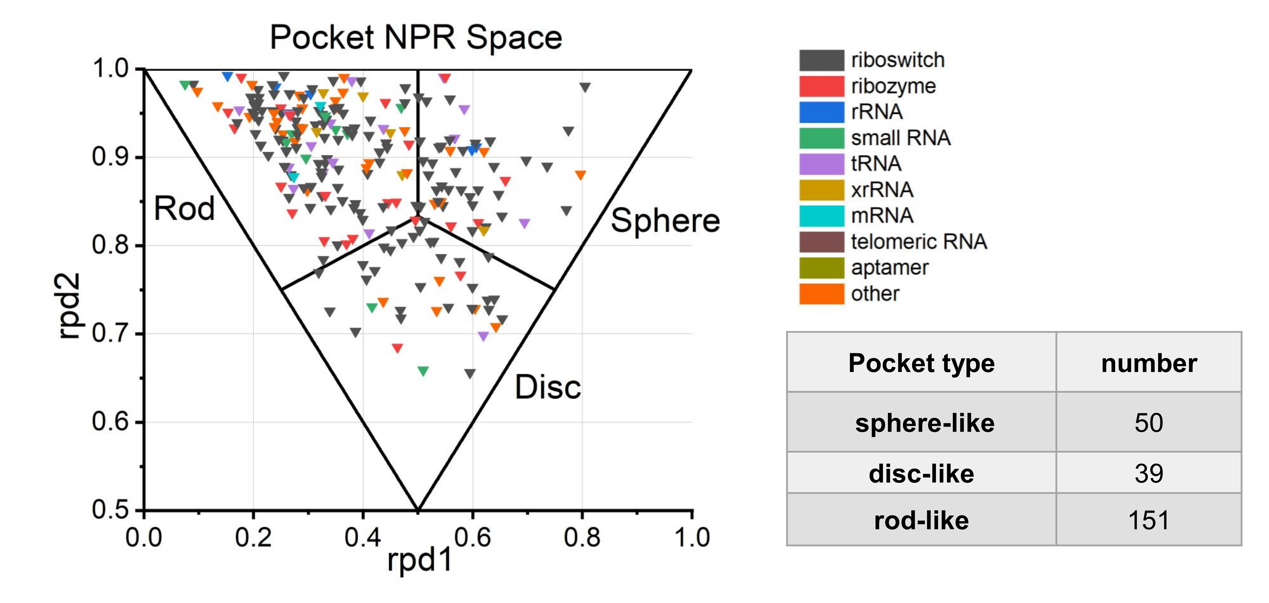
**

**Figure 3.** RNA pocket shape classification. The graph on the left shows the RNA classifications based on shape. The color reflects the RNA type of the pocket. The table shows the number of pockets for three kinds of pockets.

**
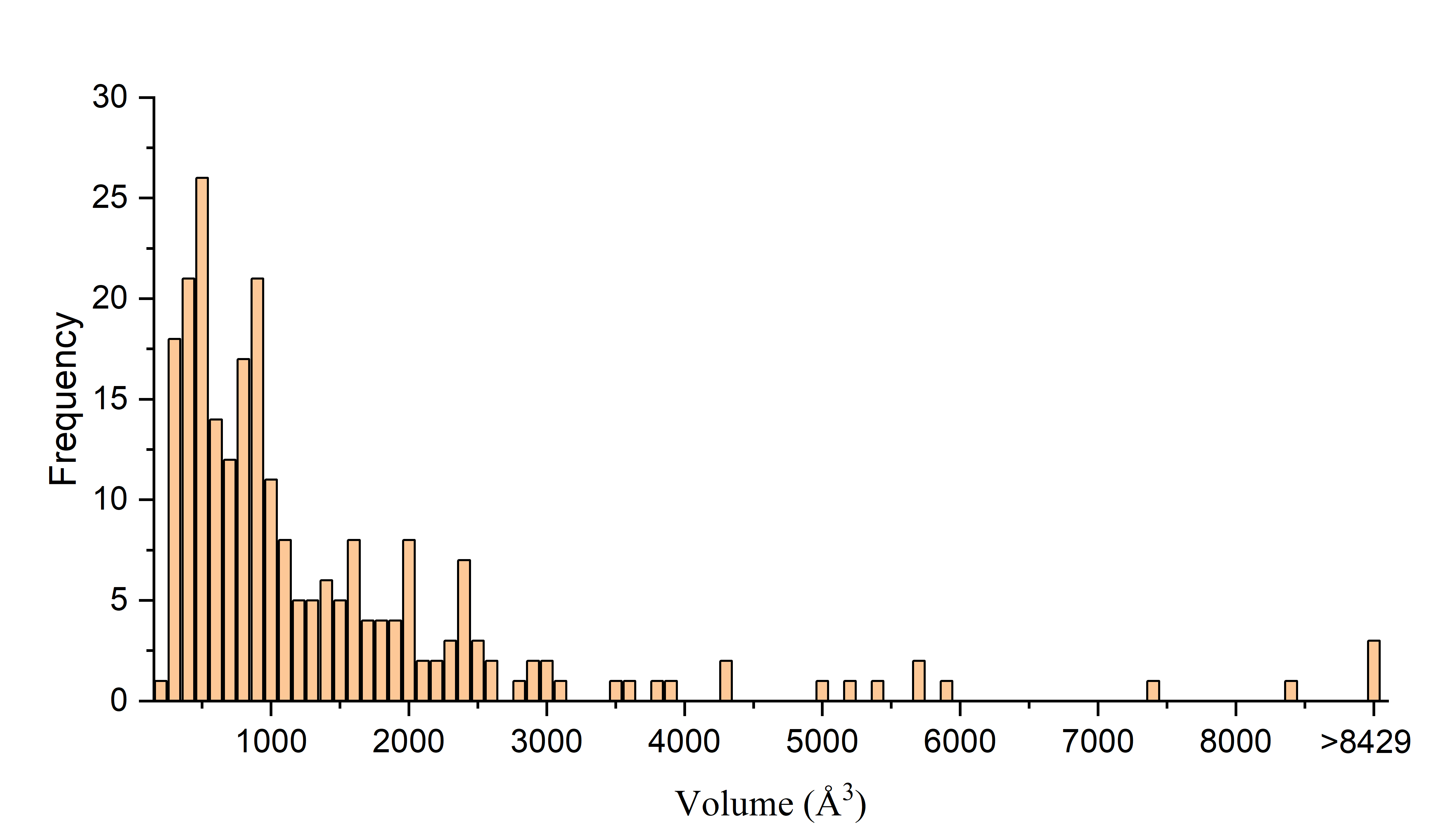
**

**Figure 4.** The volume distribution of RNA pocket. The bin size for the column is 100$Å^{3}$. For example, the column with horizontal ordinate 1000$Å^{3}$ means the frequency of pocket with the volume between 900 to 1000$Å^{3}$.

**
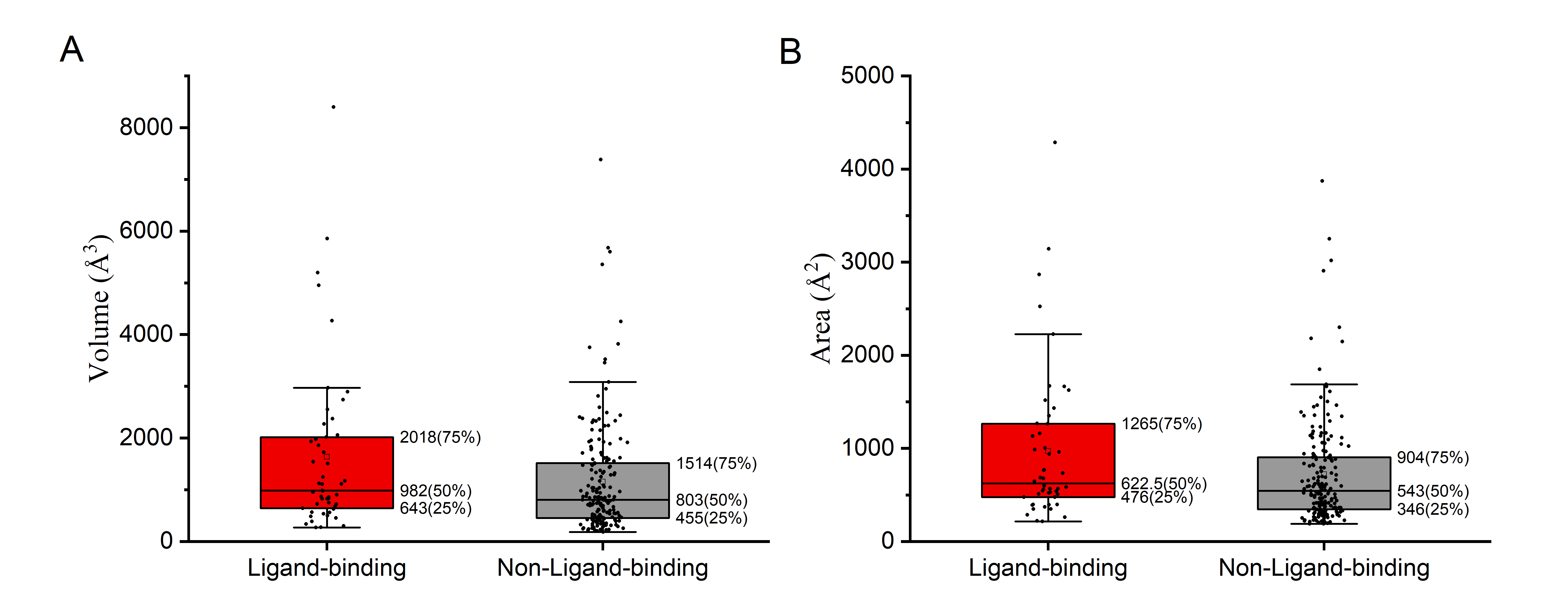
**

**Figure 5.** The distribution of (A) volume and (B) surface area for ligand-binding and non-ligand-binding pockets. The median values, ranges of the box, and their percentages are marked beside each box.


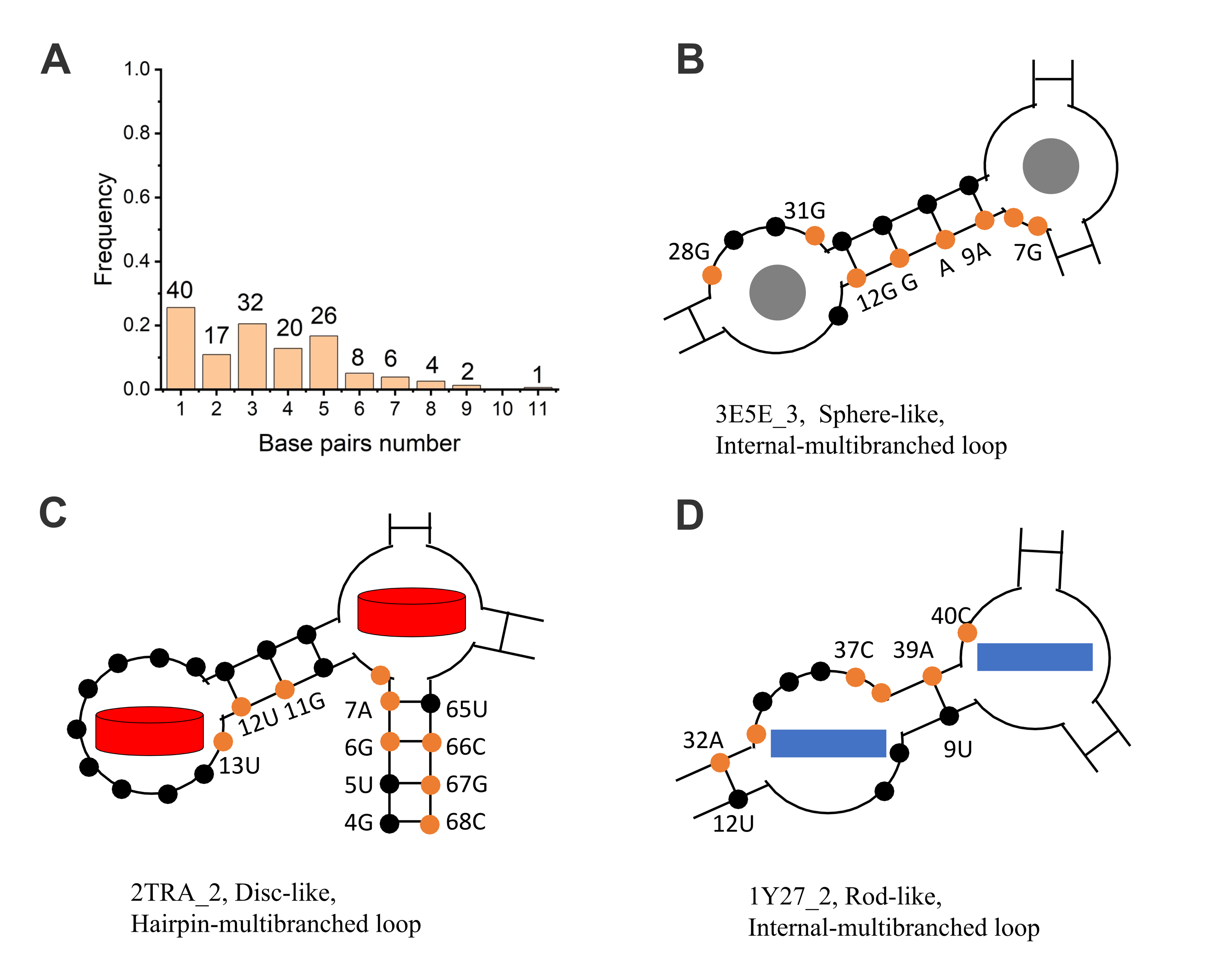


**Figure 6.** Base pairs number statistics between two adjacent loops and three examples of the secondary structure of tandem loops. (A) The statistical calculation of the base pairs between the two adjacent loops. Most of the adjacent loops are less than six base pairs (86.5%). (B-D) An example of the secondary structure of tandem loops. The tandem loops must satisfy the three conditions: (1) the two loops belonging to the same branch chain; (2) the two loops are adjacent; and (3) the distance between two loops is less than six base pairs. The sphere-like pockets are shown as gray circles. The disc-like pockets are shown as red pies. The rod-like pockets are shown as blue rectangles. The pocket binding sites are shown in orange dots.


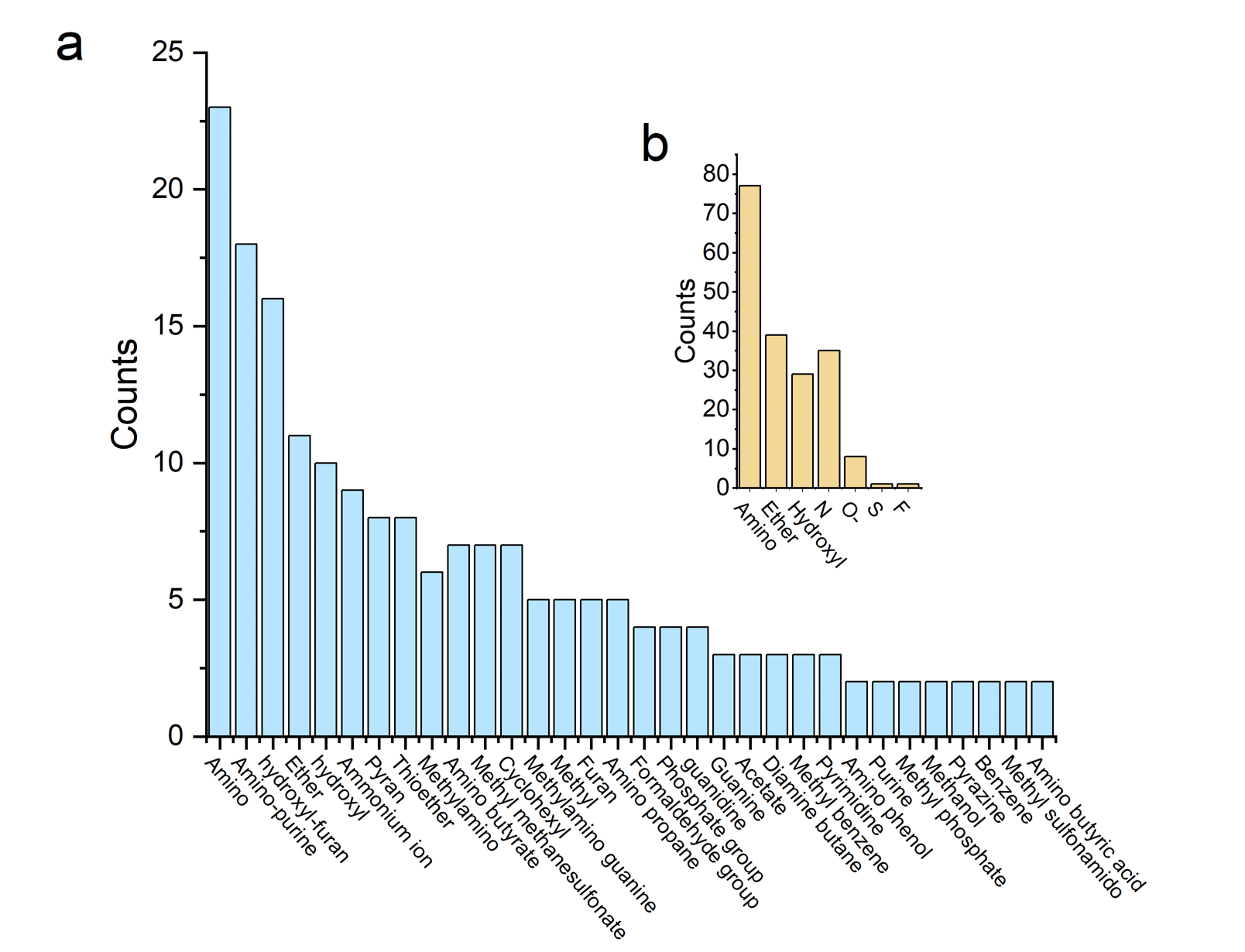


**Figure 7.** Hierarchical map of functional groups of ligands for interactions. (A) Hierarchical map of functional groups of ligands for non-bonded interactions. The functional groups involved in non-bond interactions include some bigger molecules like purine, furan, and pyran. The purine-like functional groups often form cation pi-interaction with pyrimidine of RNA. (B) Hierarchical map of functional groups of ligands for hydrogen bond interactions. The amino (77.8%), ether bond (39.4%), N (35.4%), hydroxyl (29.3%), and oxygen ion (8.1%) involve in hydrogen bond interactions. The ligands should possess electronegativity atoms to make a hydrogen bond with RNA. These various interactions can optimize the ligand and stabilize the RNA-ligand interaction.


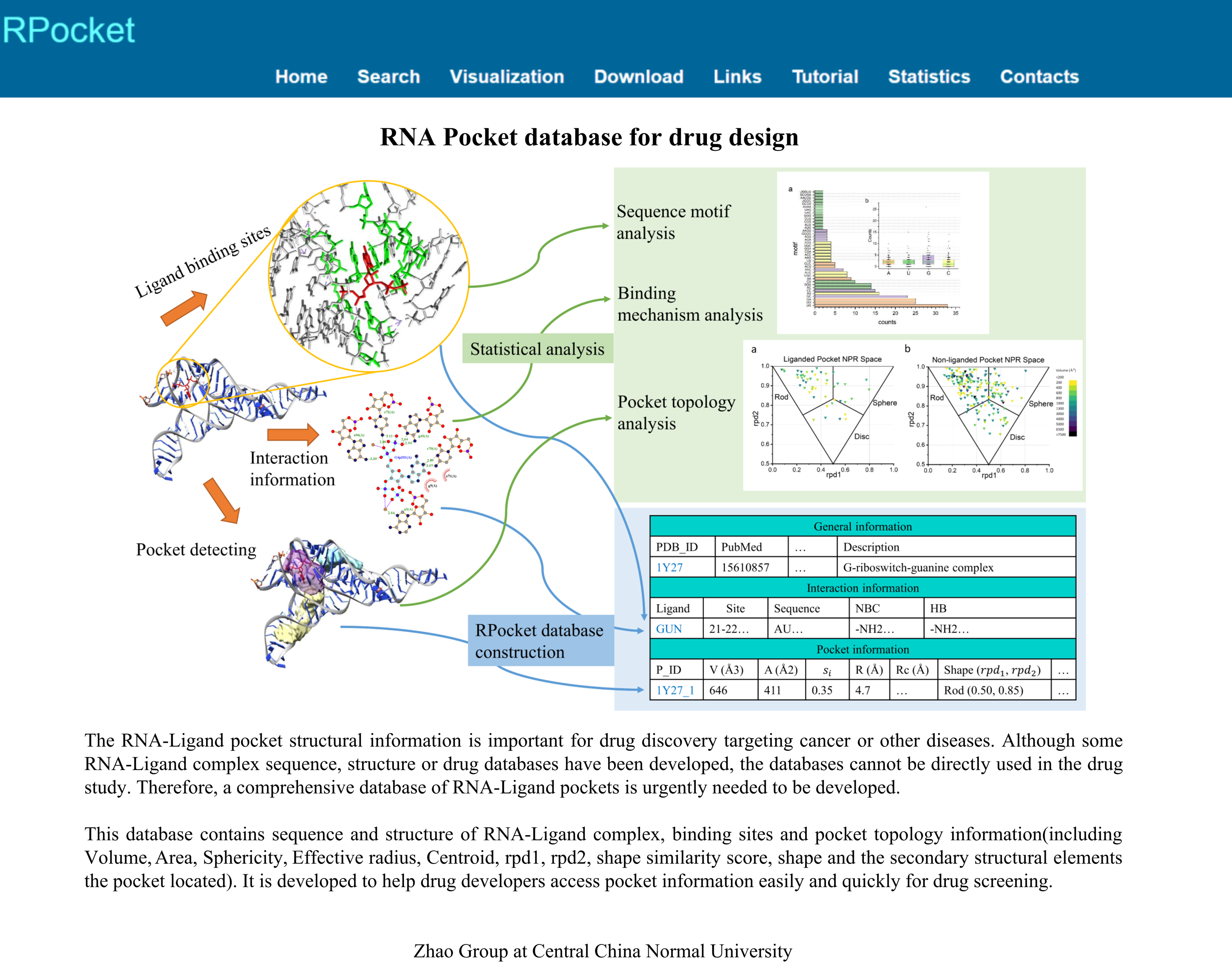


**Figure 8.** The sketch of the Home module. The Home module provides an introduction to the RPocket database and navigation to other modules of the database.


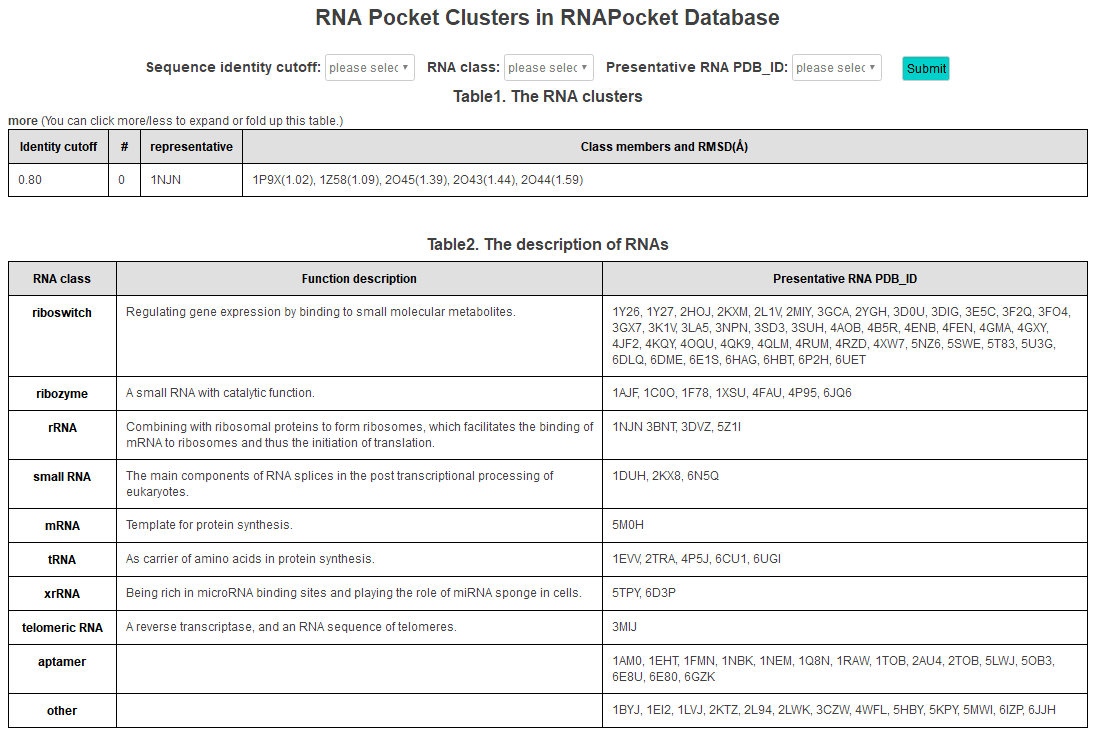


**Figure 9.** An example of the Search module. The Search module consists of a pulldown search box, a summary table of RNA clusters, and an RNA description table. (1) In the pulldown search box, users can select the object by sequence identity cutoff, RNA class, and presentative RNA PDB ID. (2) The cluster table shows the cluster conclusion of two sequence identity cutoffs, 0.80 (75 clusters) and 0.95 (94 clusters). In addition, users can expand or fold the table. (3) The RNA description table shows the representative RNAs based on their functions.


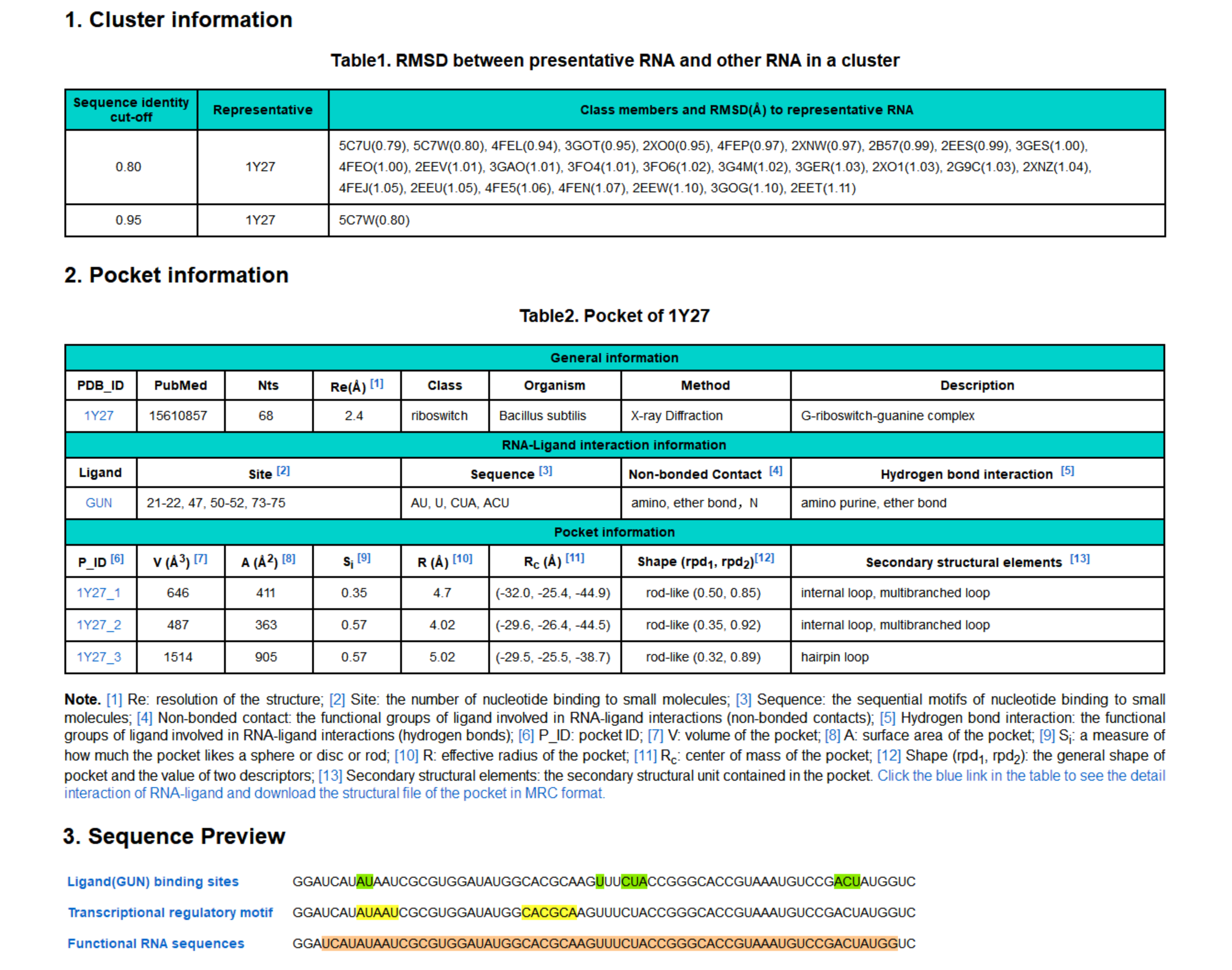


**Figure 10.** An example of RNA 1Y27 search result. The searching results including (1) The representative RNA and members in the RNA cluster; (2) the experiment information and pocket calculation results; (3) the ligand-binding sites and motifs highlighted by different colors.


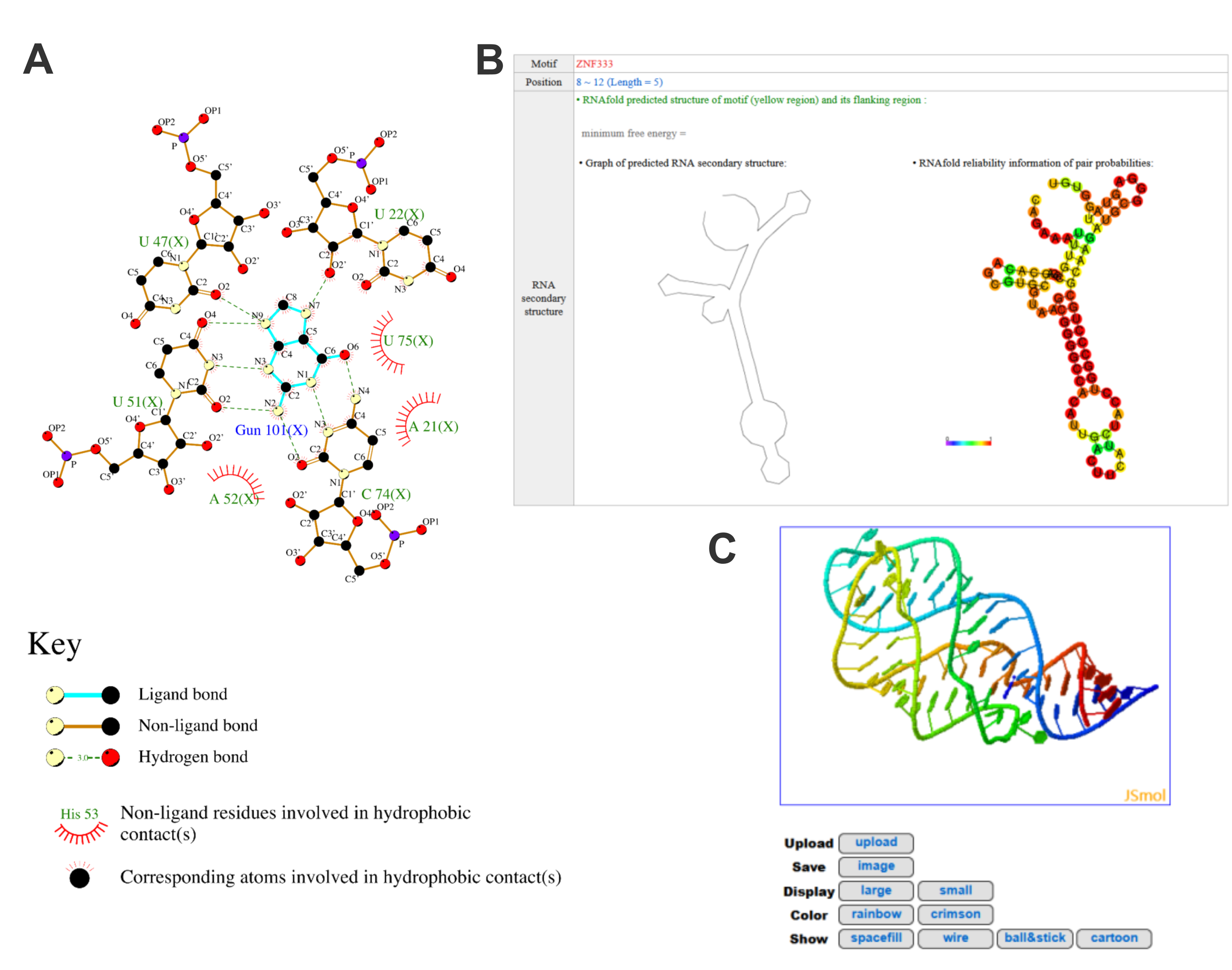


**Figure 11.** The visualization of interaction, motif, and structure. (A) The sketch of the interaction. Users can click the ligand name (colored in blue) to check the detailed interaction graph. The interaction between the ligand (GUN) and RNA (PDB: 1Y27) was generated by Ligplot+. Carbon, nitrogen, oxygen, and phosphorus atoms are colored black, cream, red, and purple. Ligand, non-ligand, and hydrogen bonds are colored by cyan, solid brown lines, and green dashed lines. Van der Waals and stacking interactions are emphasized with red arcs. (B) The sketch of the motifs. Users can click the highlight sequences in the ‘Sequence Preview’ module to enter the RegRNA server for details. The motifs were generated using the RegRNA server. (C) An example of the Visualization module. Users can scale and rotate the pocket structures. Users can also generate and save the picture.

Table 1. The comparison between RPocket and other related databases.

| **Types of databases** | **Databases** | **Links** | **RNA-ligand complex** | | **Interaction information** | | | | **Pocket information** | | | **Statistical analysis** | **Docking benchmark or function** |
| --- | --- | --- | --- | --- | --- | --- | --- | --- | --- | --- | --- | --- | --- |
|  |  |  | **RNA structure** | **Ligand physicochemical property** | **Binding site** | **Secondary elements** | **Interaction graph** | **kinetic data** | **Structure** | **Size** | **Shape** |  |  |
| RNA-ligand structure databases | PDB | [https://www.rcsb.org](https://www.rcsb.org/) | ✔ | ✔ |  |  |  |  |  |  |  |  |  |
|  | NAD | <http://ndbserver.rutgers.edu/> | ✔ |  |  |  |  |  |  |  |  |  |  |
|  | PDB-Ligand | http://www.idrtech.com/PDB-Ligand/ | ✔ |  |  |  |  |  |  |  |  | ✔ |  |
|  | R-bind | [https://rbind.chem.duke.edu](https://rbind.chem.duke.edu/) |  | ✔ |  | ✔ |  | ✔ |  |  |  | ✔ |  |
| RNA-ligand experimental databases | NALDB | http://bsbe.iiti.ac.in/bsbe/naldb/HOME.php |  | ✔ | ✔ |  |  | ✔ |  |  |  |  |  |
|  | SMMRNA | [http://www.smmrna.org](http://www.smmrna.org/) | ✔ | ✔ |  |  |  | ✔ |  |  |  |  |  |
|  | KDBI | http:// xin.cz3.nus.edu.sg/group/kdbi/kdbi.asp | ✔ |  |  |  |  | ✔ |  |  |  |  |  |
| RNA docking datasets and tools | RRDB | <http://huanglab.phys.hust.edu.cn/RRDbenchmark/> |  |  |  |  |  |  |  |  |  |  | RNA-RNA |
|  | HNADOCK | http://huanglab.phys.hust.edu.cn/hnadock/ |  |  |  |  |  |  |  |  |  |  | RNA-RNA |
|  | DrugScoreRNA |  |  |  |  |  |  |  |  |  |  |  | function |
|  | LigandRNA | [http://ligandrna.genesilico.pl](http://ligandrna.genesilico.pl/) |  |  |  |  |  |  |  |  |  |  | RNA-ligand, function |
| RNA pocket databases and tools | 3V | http://3vee.molmovdb.org/ |  |  |  |  |  |  | ✔ | ✔ |  |  |  |
|  | Caver | https://www.caver.cz/ |  |  |  |  |  |  | ✔ |  |  |  |  |
|  | PocketFinder | <http://www.bioinformatics.leeds.ac.uk/pocketfinder> |  |  |  |  |  |  | ✔ | ✔ |  |  |  |
|  | Rpocket | <http://zhaoserver.com.cn/RPocket/RPocket.html> | ✔ |  | ✔ | ✔ | ✔ |  | ✔ | ✔ | ✔ | ✔ |  |

**References**

1. Berman H, Westbrook J, Feng Z, Gilliland G, Bhat T, Weissig H, Shindyalov I, Bourne P: **The Protein Data Bank**. *Nucleic Acids Research* 2000, **28**(1):235-242.

2. Berman HM, Olson WK, Beveridge DL, Westbrook J, Schneider B: **The nucleic acid database. A comprehensive relational database of three-dimensional structures of nucleic acids**. *Biophysical Journal* 1992, **63**(3):751-759.

3. Jae-Min S, Doo-Ho C: **PDB-Ligand: a ligand database based on PDB for the automated and customized classification of ligand-binding structures**. *Nucleic Acids Research* 2005, **33**:D238-D241.

4. Morgan BS, Sanaba BG, Donlic A, Karloff DB, Hargrove AE: **R-BIND: An Interactive Database for Exploring and Developing RNA-Targeted Chemical Probes**. *ACS Chemical Biology* 2019, **14**(12):2691–2700.

5. Subodh, Kumar, Mishra, Amit, Kumar: **NALDB: nucleic acid ligand database for small molecules targeting nucleic acid**. *Database* 2016, **2016**:1-11.

6. Ankita M, Surabhi S, Isha G, Saurabh L, Sharma DK, Raman P: **SMMRNA: a database of small molecule modulators of RNA**. *Nucleic Acids Research* 2014, **42**(D1):132-141.

7. Ji ZL, Chen X, Zhen CJ, Yao LX, Han LY, Yeo WK, Chung PC, Puy HS, Tay YT, Muhammad A: **KDBI: Kinetic Data of Bio-molecular Interactions database**. *Nucleic Acids Research* 2003, **31**(1):255-257.

8. Yan Y, Sheng-You H: **RRDB: A comprehensive and nonredundant benchmark for RNA-RNA docking and scoring**. *Bioinformatics* 2017, **34**(3):453-458.

9. He J, Wang J, Tao H, Xiao Y, Huang SY: **HNADOCK: a nucleic acid docking server for modeling RNA/DNA-RNA/DNA 3D complex structures**. *Nucleic acids research* 2019, **47**:W35-W42.

10. Pfeffer P, Gohlke H: **DrugScoreRNA Knowledge-Based Scoring Function To Predict RNA-Ligand Interactions**. *Journal of Chemical Information and Modeling* 2007, **47**(5):1868-1876.

11. Philips A, Milanowska K, Lach G, Bujnicki JM: **LigandRNA: computational predictor of RNA-ligand interactions**. *Rna* 2013, **19**(12):1605-1616.

12. Voss NR, Gerstein M: **3V: cavity, channel and cleft volume calculator and extractor**. *Nucleic Acids Research* 2010, **38**:W555-W562.

13. Petřek M, Otyepka, M., Banáš, P., Košinová, P., Koča, J., Damborský, J.: **CAVER: a new tool to explore routes from protein clefts, pockets and cavities**. *Bmc Bioinformatics* 2006, **7**(1):316.

14. **Pocketome via Comprehensive Identification and Classification of Ligand Binding Envelopes**. *Molecular & Cellular Proteomics* 2005, **4**(6):752-761.

15. Guilloux VL, Schmidtke P, Tuffery P: **Fpocket: An open source platform for ligand pocket detection**. *Bmc Bioinformatics* 2009, **10**(1):1-11.

16. Lam JH, Li Y, Zhu L, Umarov R, Gao X: **A deep learning framework to predict binding preference of RNA constituents on protein surface**. *Nature Communications* 2019, **10**(1).
