# Supplementary material for: RPocket: an intuitive database of RNA pocket topology information with RNA-ligand data resources
Source: BMC Bioinformatics. 2021 Sep 8;22:428. doi: 10.1186/s12859-021-04349-4 (PMC8424408; doi:10.1186/s12859-021-04349-4)
Supplement: Supplementary file 6 — Additional file 6. Folder S2: Interaction info of RNA-ligand complexes. [file 12859_2021_4349_MOESM6_ESM.zip › 12859_2021_4349_MOESM6_ESM/1AM0/1AM0.pdf]

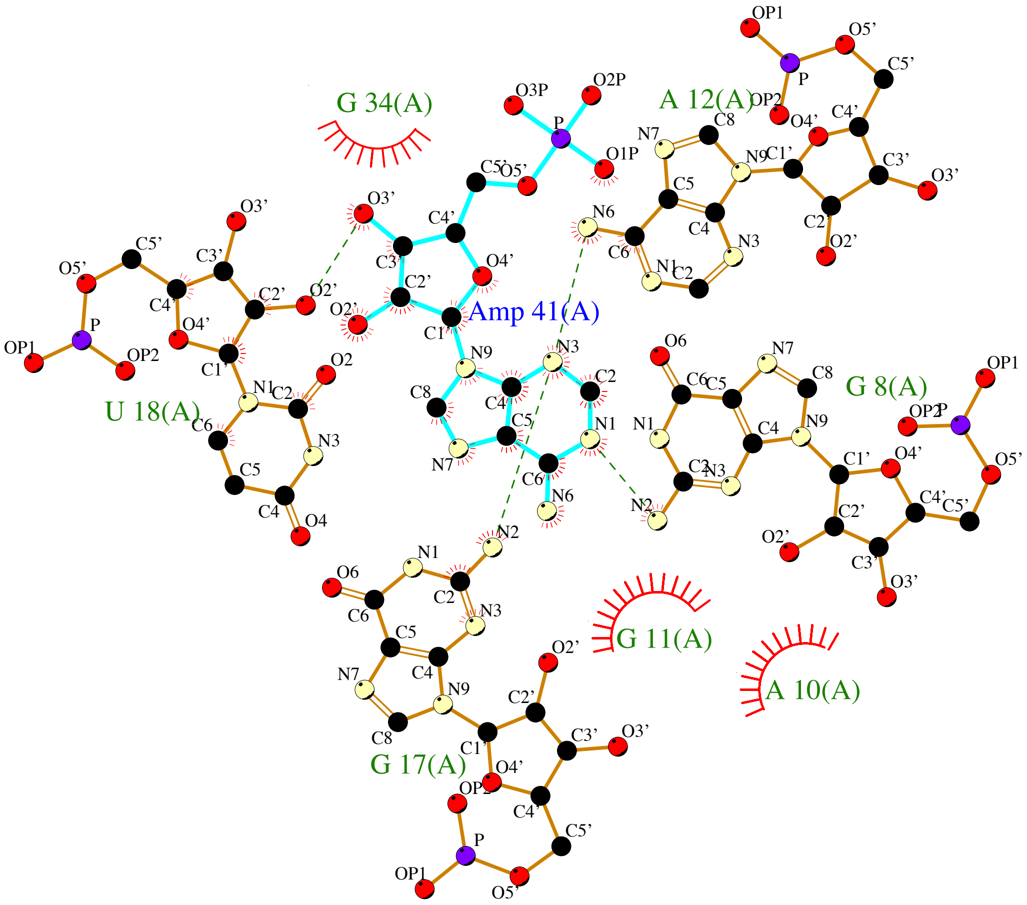

G 34(A)
A 12(A)
Amp 41(A)
U 18(A)
G 8(A)
G 11(A)
A 10(A)
G 17(A)

Key

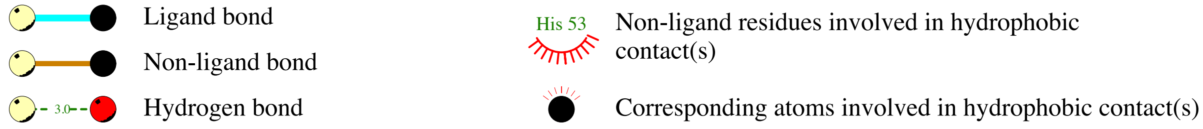

Ligand bond
Non-ligand bond
3.0
Hydrogen bond
His 53
Non-ligand residues involved in hydrophobic contact(s)
Corresponding atoms involved in hydrophobic contact(s)

ligplus
