# Supplementary material for: RPocket: an intuitive database of RNA pocket topology information with RNA-ligand data resources
Source: BMC Bioinformatics. 2021 Sep 8;22:428. doi: 10.1186/s12859-021-04349-4 (PMC8424408; doi:10.1186/s12859-021-04349-4)
Supplement: Supplementary file 6 — Additional file 6. Folder S2: Interaction info of RNA-ligand complexes. [file 12859_2021_4349_MOESM6_ESM.zip › 12859_2021_4349_MOESM6_ESM/1BYJ/1BYJ.pdf]

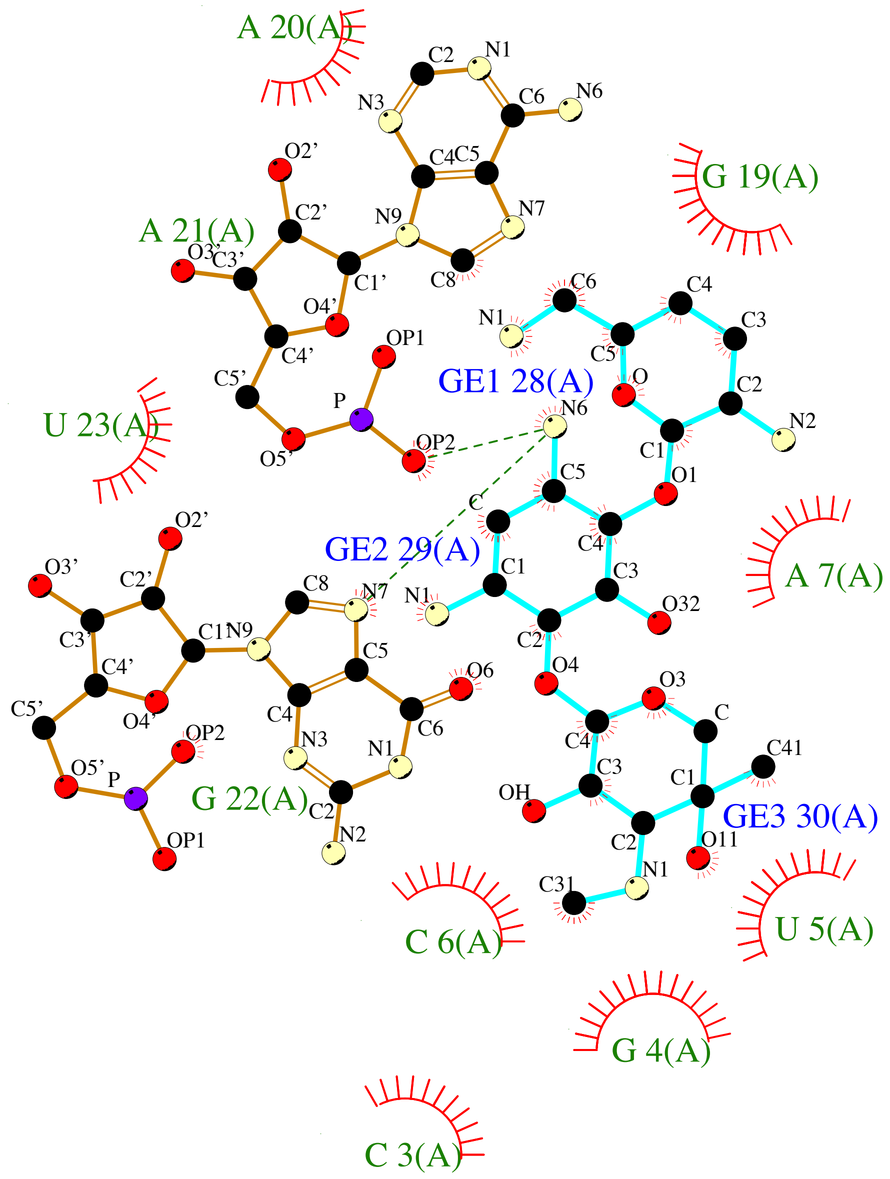

A 20(A)
A 21(A)
G 19(A)
GE1 28(A)
U 23(A)
GE2 29(A)
A 7(A)
G 22(A)
GE3 30(A)
C 6(A)
U 5(A)
G 4(A)
C 3(A)

Key

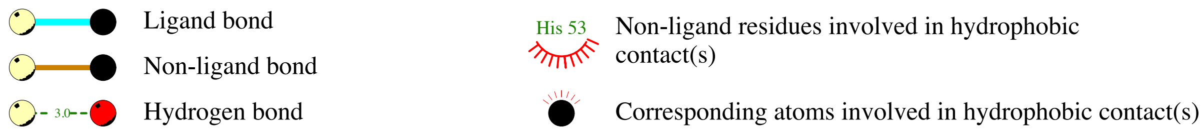

Ligand bond
Non-ligand bond
3.0
Hydrogen bond
His 53
Non-ligand residues involved in hydrophobic contact(s)
Corresponding atoms involved in hydrophobic contact(s)

ligplus
