# Supplementary material for: RPocket: an intuitive database of RNA pocket topology information with RNA-ligand data resources
Source: BMC Bioinformatics. 2021 Sep 8;22:428. doi: 10.1186/s12859-021-04349-4 (PMC8424408; doi:10.1186/s12859-021-04349-4)
Supplement: Supplementary file 6 — Additional file 6. Folder S2: Interaction info of RNA-ligand complexes. [file 12859_2021_4349_MOESM6_ESM.zip › 12859_2021_4349_MOESM6_ESM/1EVV/1EVV.pdf]

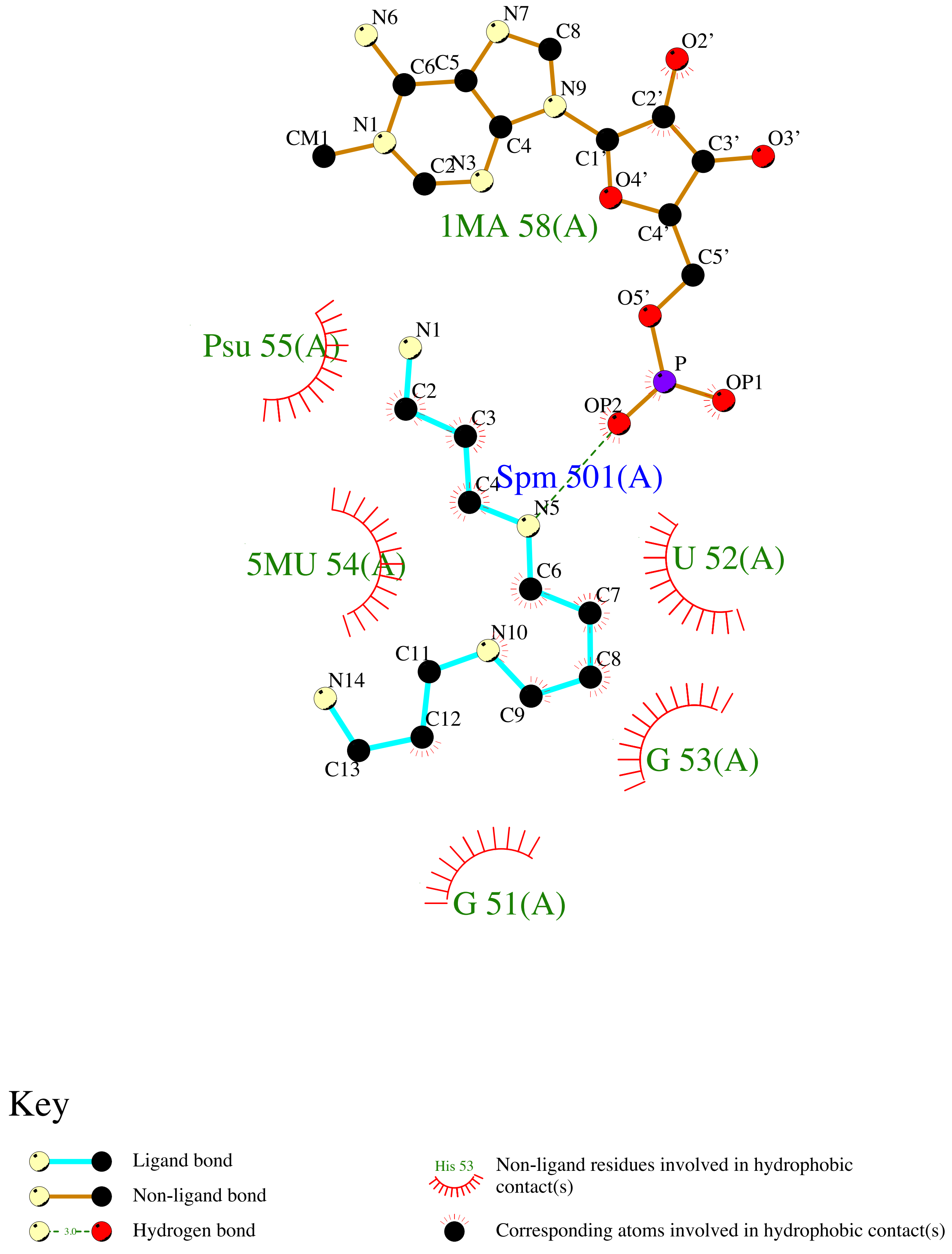
N6
N7
C8
O2'
C6C5
N9
C2'
N1
CM1
C3'
O3'
C4
C1'
C2N3
O4'
1MA 58(A)
C4'
C5'
O5'
Psu 55(A)
N1
P
C2
OP1
OP2
C3
Spm 501(A)
C4
N5
5MU 54(A)
U 52(A)
C6
C7
N10
C11
C8
N14
C12
C9
C13
G 53(A)
G 51(A)
Key
Ligand bond
Non-ligand bond
3.0
Hydrogen bond
His 53
Non-ligand residues involved in hydrophobic contact(s)
Corresponding atoms involved in hydrophobic contact(s)

ligplus
