# Supplementary material for: RPocket: an intuitive database of RNA pocket topology information with RNA-ligand data resources
Source: BMC Bioinformatics. 2021 Sep 8;22:428. doi: 10.1186/s12859-021-04349-4 (PMC8424408; doi:10.1186/s12859-021-04349-4)
Supplement: Supplementary file 6 — Additional file 6. Folder S2: Interaction info of RNA-ligand complexes. [file 12859_2021_4349_MOESM6_ESM.zip › 12859_2021_4349_MOESM6_ESM/1XSU/1XSU.pdf]

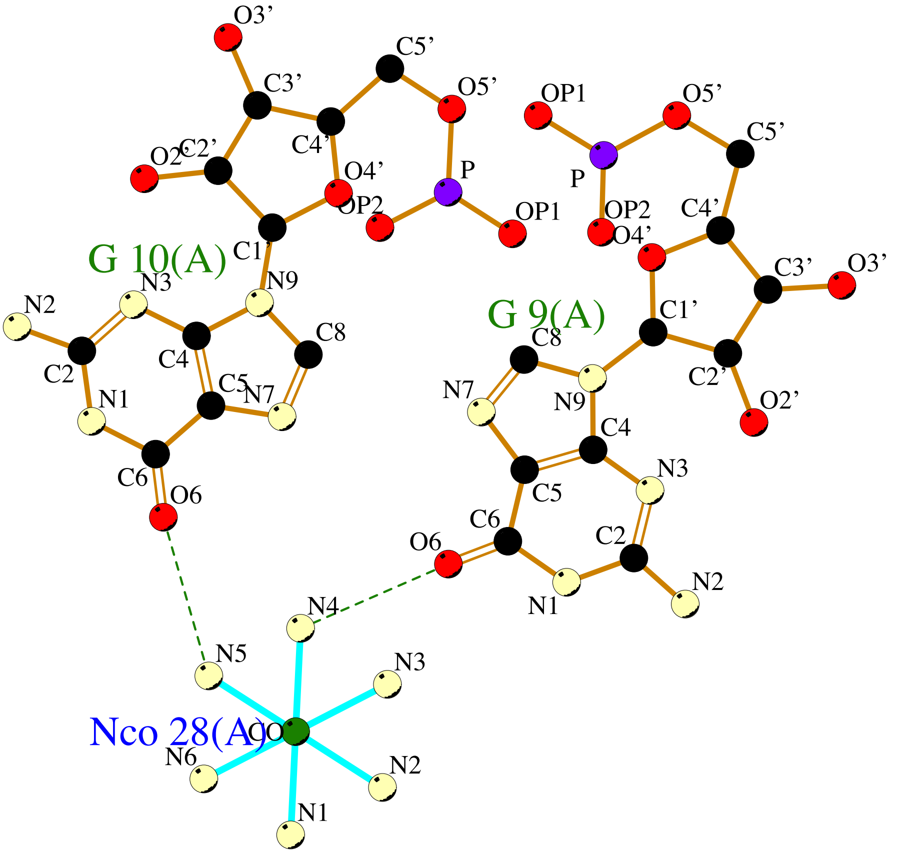

Key

Ligand bond

Non-ligand bond

3.0 Hydrogen bond

His 53 Non-ligand residues involved in hydrophobic contact(s)

Corresponding atoms involved in hydrophobic contact(s)

ligplus
