# Supplementary material for: RPocket: an intuitive database of RNA pocket topology information with RNA-ligand data resources
Source: BMC Bioinformatics. 2021 Sep 8;22:428. doi: 10.1186/s12859-021-04349-4 (PMC8424408; doi:10.1186/s12859-021-04349-4)
Supplement: Supplementary file 6 — Additional file 6. Folder S2: Interaction info of RNA-ligand complexes. [file 12859_2021_4349_MOESM6_ESM.zip › 12859_2021_4349_MOESM6_ESM/2TRA/2TRA.pdf]

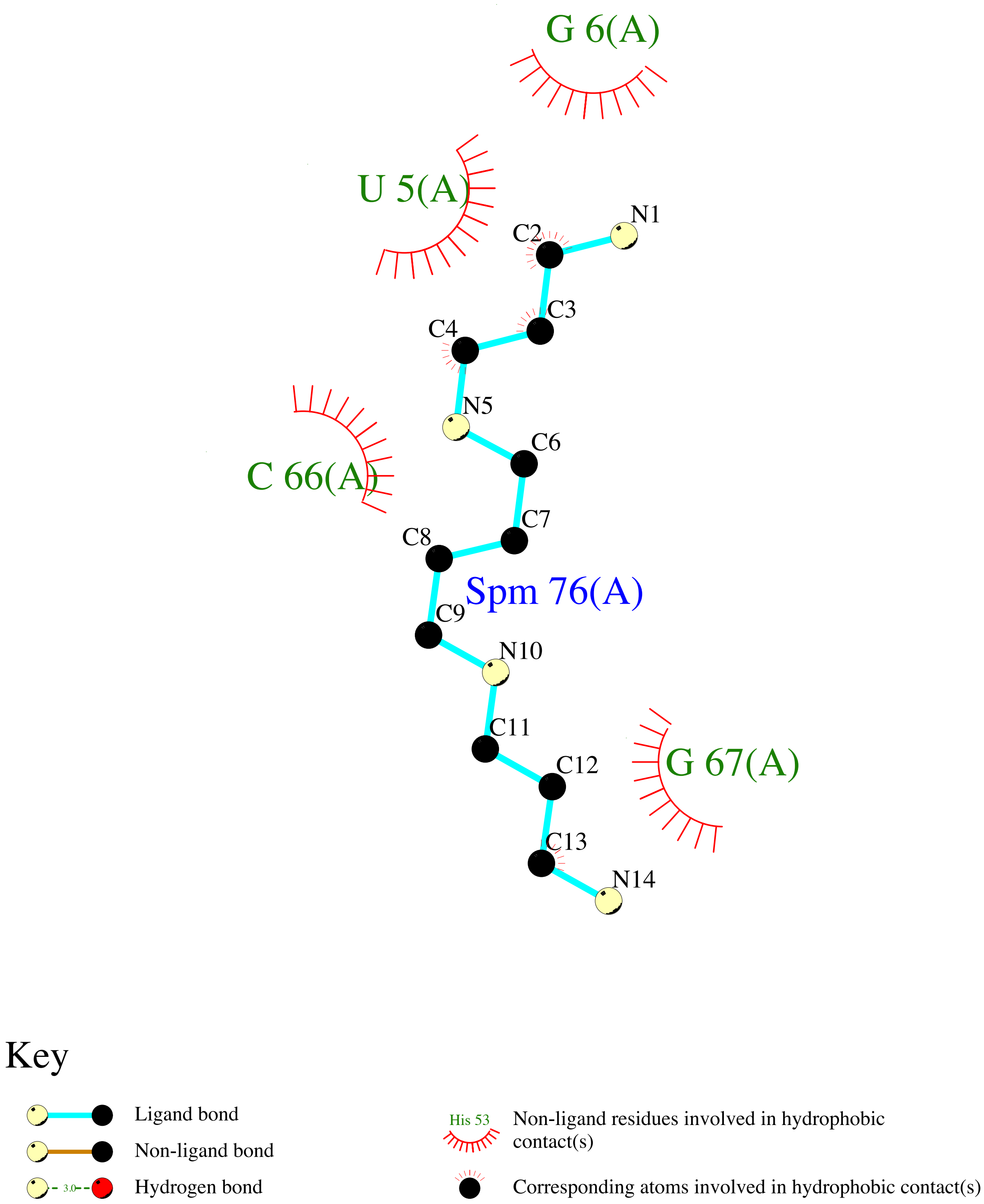
G 6(A)
U 5(A)
N1
C2
C3
C4
N5
C6
C 66(A)
C7
C8
Spm 76(A)
C9
N10
C11
C12
G 67(A)
C13
N14
Key
Ligand bond
Non-ligand bond
3.0
Hydrogen bond
His 53
Non-ligand residues involved in hydrophobic contact(s)
Corresponding atoms involved in hydrophobic contact(s)

ligplus
