# Supplementary material for: RPocket: an intuitive database of RNA pocket topology information with RNA-ligand data resources
Source: BMC Bioinformatics. 2021 Sep 8;22:428. doi: 10.1186/s12859-021-04349-4 (PMC8424408; doi:10.1186/s12859-021-04349-4)
Supplement: Supplementary file 6 — Additional file 6. Folder S2: Interaction info of RNA-ligand complexes. [file 12859_2021_4349_MOESM6_ESM.zip › 12859_2021_4349_MOESM6_ESM/4FEN_NCO/4FEN_NCO.pdf]

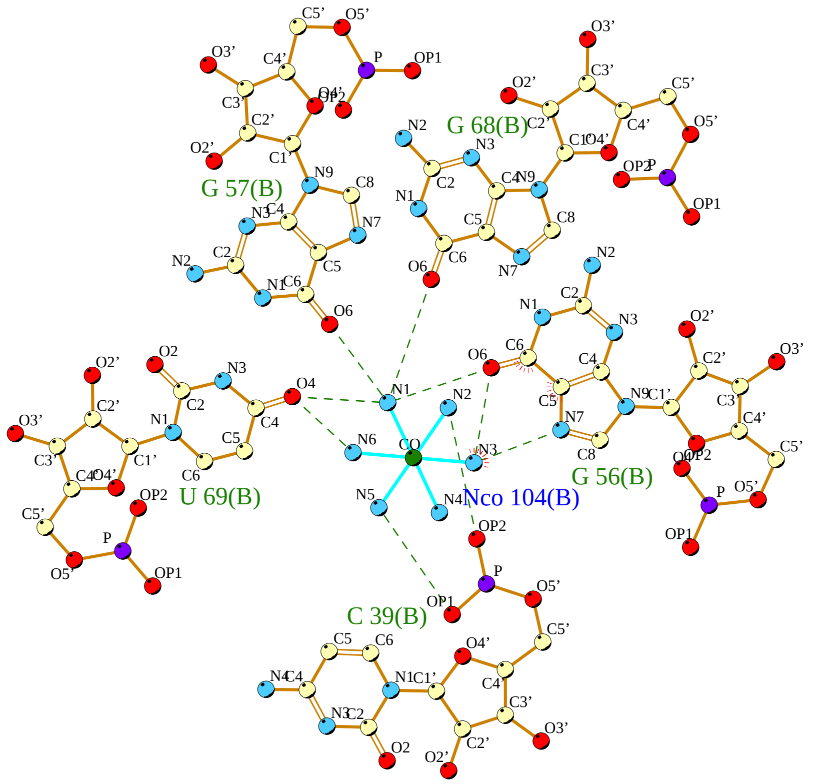

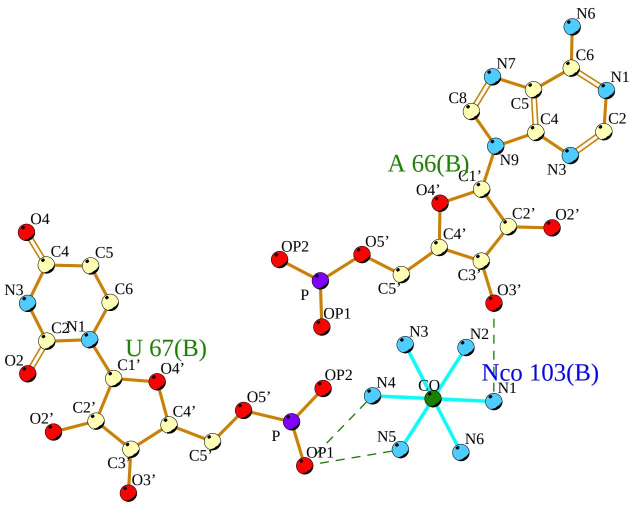

Key

Ligand bond	His 53 — Non-ligand residues involved in hydrophobic contact(s)
Non-ligand bond	
3.0 Hydrogen bond	Corresponding atoms involved in hydrophobic contact(s)

ligplus
